# The Small RNA GcvB Promotes Mutagenic Break Repair by Opposing the Membrane Stress Response

**DOI:** 10.1128/JB.00555-16

**Published:** 2016-11-18

**Authors:** Brittany Barreto, Elizabeth Rogers, Jun Xia, Ryan L. Frisch, Megan Richters, Devon M. Fitzgerald, Susan M. Rosenberg

**Affiliations:** a Department of Molecular and Human Genetics, Baylor College of Medicine, Houston, Texas, USA; b Department of Biochemistry and Molecular Biology, Baylor College of Medicine, Houston, Texas, USA; c Department of Molecular Virology and Microbiology, Baylor College of Medicine, Houston, Texas, USA; d Dan L. Duncan Comprehensive Cancer Center, Baylor College of Medicine, Houston, Texas, USA; Ohio State University

## Abstract

Microbes and human cells possess mechanisms of mutagenesis activated by stress responses. Stress-inducible mutagenesis mechanisms may provide important models for mutagenesis that drives host-pathogen interactions, antibiotic resistance, and possibly much of evolution generally. In Escherichia coli, repair of DNA double-strand breaks is switched to a mutagenic mode, using error-prone DNA polymerases, via the SOS DNA damage and general (σ^S^) stress responses. We investigated small RNA (sRNA) clients of Hfq, an RNA chaperone that promotes mutagenic break repair (MBR), and found that GcvB promotes MBR by allowing a robust σ^S^ response, achieved via opposing the membrane stress (σ^E^) response. Cells that lack *gcvB* were MBR deficient and displayed reduced σ^S^-dependent transcription but not reduced σ^S^ protein levels. The defects in MBR and σ^S^-dependent transcription in Δ*gcvB* cells were alleviated by artificially increasing σ^S^ levels, implying that GcvB promotes mutagenesis by allowing a normal σ^S^ response. Δ*gcvB* cells were highly induced for the σ^E^ response, and blocking σ^E^ response induction restored both mutagenesis and σ^S^-promoted transcription. We suggest that GcvB may promote the σ^S^ response and mutagenesis indirectly, by promoting membrane integrity, which keeps σ^E^ levels lower. At high levels, σ^E^ might outcompete σ^S^ for binding RNA polymerase and so reduce the σ^S^ response and mutagenesis. The data show the delicate balance of stress response modulation of mutagenesis.

**IMPORTANCE** Mutagenesis mechanisms upregulated by stress responses promote *de novo* antibiotic resistance and cross-resistance in bacteria, antifungal drug resistance in yeasts, and genome instability in cancer cells under hypoxic stress. This paper describes the role of a small RNA (sRNA) in promoting a stress-inducible-mutagenesis mechanism, mutagenic DNA break repair in Escherichia coli. The roles of many sRNAs in E. coli remain unknown. This study shows that Δ*gcvB* cells, which lack the GcvB sRNA, display a hyperactivated membrane stress response and reduced general stress response, possibly because of sigma factor competition for RNA polymerase. This results in a mutagenic break repair defect. The data illuminate a function of GcvB sRNA in opposing the membrane stress response, and thus indirectly upregulating mutagenesis.

## INTRODUCTION

Bacterial (1–7), yeast (8), and human cancer (9, 10) cells possess mechanisms of mutagenesis upregulated by stress responses. Stress-inducible mutation mechanisms may accelerate adaptation specifically when cells are poorly adapted to their environments, i.e., when stressed. Modeling studies indicate that stress-inducible mutagenesis can be selected on the basis of acceleration of adaptation even in asexual bacterial populations, in which deleterious mutations generated cannot be purged by recombination (11, 12). Stress-inducible mutation mechanisms drive evolution of antibiotic resistance (13–15) and cross-resistance (16), antifungal drug resistance (8, 17) and possibly much of evolution generally.

In Escherichia coli, repair of DNA double-strand breaks (DSBs) by homologous recombination is switched to a mutagenic mode using error-prone DNA polymerases under the control of the SOS DNA damage response and the general stress response (1, 2, 6, 18–20). Mutagenic break repair (MBR) requires proteins that perform DSB repair via homologous recombination (21–23), low-fidelity DNA polymerases (Pols) IV (18, 19, 24), V (19, 25), and II (26), and the activators of the general/starvation stress response (RpoS/σ^S^) (18, 19, 27, 28), the SOS DNA damage response (29, 30), and the RpoE/σ^E^ membrane stress response (31). The σ^E^ response drives mutagenesis by promoting spontaneous DNA breakage (31) at some genomic locations, as do RNA/DNA hybrids (R-loops) caused by transcription (32). The SOS response promotes mutagenesis via its 10-fold transcriptional upregulation of Pol IV (33) and by allowing production of Pol V. The general stress response is activated by the σ^S^ transcriptional activator, a sigma factor of RNA polymerase, in response to starvation, antibiotics (16), and many other stresses (34, 35). The general stress response directly and indirectly up- and downregulates transcription of more than 500 E. coli genes (34, 35) and promotes mutagenesis by allowing the use of, or errors made by, the error-prone DNA Pols in DSB repair, by an as-yet-unknown mechanism (18, 19, 26). Thus, even in cells with a DSB, an activated SOS response and the resulting 10-fold-higher levels of Pol IV, DSB repair remains relatively nonmutagenic, using high-fidelity DNA Pol III (36), unless the general stress response is also activated either by stress or artificially (18, 19, 26). That is, σ^S^-inducing stress is not itself needed for mutagenesis during DSB repair; artificial activation of the σ^S^ response is sufficient to make repair mutagenic even in growing cells (18, 19). The MBR mechanism, in which DNA Pol IV initiates mutagenic DNA synthesis from a D-loop (intermediate in recombinational repair), has been recapitulated in solution with purified proteins (37). Further, the mutation signatures of σ^S^-promoted mutagenesis are overrepresented in extant bacterial genomes, suggesting that MBR is widespread in bacterial mutagenesis in the wild (38).

Mutagenic break repair in E. coli is promoted by a large network of more than 93 genes, mutations in any of which decrease mutagenesis (39). More than half of MBR network genes promote mutagenesis by sensing stress and transducing signals that lead to activation of the σ^S^, SOS, and/or σ^E^ stress responses (39), indicating the importance of stress response control of mutagenesis to E. coli. Among the genes discovered in this screen for MBR-defective mutants is *hfq*, which encodes the Hfq RNA chaperone (39). Hfq is required for MBR in E. coli (39).

Hfq was discovered as a bacterial host factor required for synthesis of bacteriophage Q_β_ RNA (40) and is part of the conserved family of Sm-like RNA-modulating proteins found in eukaryotes, archaea, and eubacteria (41). Hfq is required for virulence of several bacterial species (42–49). Acting as an RNA chaperone, Hfq facilitates base pairing of a collection of small RNAs (sRNAs) to specific mRNA molecules, which allows the sRNAs to up- or-downregulate translation from the mRNAs (50, 51). sRNAs are approximately 100 bp long and downregulate translation of some mRNAs by base pairing that blocks ribosome-binding sites (52). sRNAs also upregulate translation by melting mRNA secondary structures such as hairpins that would otherwise prevent ribosome recognition (53). Several sRNAs are upregulated during stress, including DsrA and RprA, both of which promote translation of the *rpoS* mRNA to σ^S^ protein (54). Of the approximately 100 sRNAs known in E. coli (55–58), 30 sRNAs require Hfq to function (59). Although the means by which Hfq promotes MBR is unknown, the fact that it does so suggests that one or more of the Hfq client sRNAs may promote mutagenesis. In this study, we examined nine sRNA clients of Hfq that are not encoded within protein-coding genes and that showed expression patterns potentially relevant to starvation stress (59). We report below that cells that lack the GcvB sRNA are MBR defective.

Found in diverse bacteria, GcvB is an Hfq-chaperoned sRNA that up- or downregulates translation of amino acid biosynthesis and transport proteins (60–63). In Salmonella enterica, GcvB is a master regulator of amino acid metabolism and directly up- or downregulates translation of ∼1% of all mRNAs (64). GcvB regulates a network of mRNAs by inhibiting or enabling translation based on cellular environment. E. coli Δ*gcvB* mutant cells are acid sensitive, possibly caused partly by reduced σ^S^ levels, shown with a σ^S^-LacZ fusion protein (65).

Many sRNAs in E. coli promote membrane integrity and do so by regulating outer membrane protein genes (66–68). The levels of various sRNAs are increased under different stresses (56, 69), and many are upregulated by the σ^E^ membrane stress response (70–72). sRNAs, such as MicA, RybB, and MicL, are induced by cell envelope stress, and then they downregulate translation of outer membrane porins and lipoproteins, aiding membrane integrity (73–77). Transcription of *rpoE* and σ^E^-dependent promoter use is increased in an *hfq* mutant, supporting the roles of sRNAs in averting the σ^E^ response and promoting membrane integrity (78). Here, we show that the GcvB sRNA is required for MBR. We find that GcvB promotes MBR by allowing a robust σ^S^ response. We report that Δ*gcvB* mutant cells display reduced σ^S^-regulated promoter activity and MBR but not reduced σ^S^ protein levels. Artificial upregulation of σ^S^ restored σ^S^-regulated promoter activity and MBR, implying that normal quantities of σ^S^ are insufficient to activate the general stress response in Δ*gcvB* cells. We provide evidence that the MBR and σ^S^ response deficiency in Δ*gcvB* cells result from hyperactivation of the σ^E^ membrane stress response. We suggest that GcvB may promote the σ^S^ response, and so also MBR, indirectly by keeping membrane stress low enough for σ^S^ to compete successfully with σ^E^ for RNA polymerase. The data illuminate a function of GcvB in opposing the membrane stress response, and thus indirectly upregulating mutagenesis.

## MATERIALS AND METHODS

### Strains and materials.

E. coli K-12 strains and plasmids used are shown in Table 1. Strains were constructed using phage lambda Red-mediated recombineering as described previously (79) and phage P1-mediated transduction as described previously (80). M9 minimal medium (80) had carbon sources added at 0.1% and vitamin B_1_ (B1) at 10 μg/ml. LBH medium was as described previously (81). Antibiotics and other additives were used at the following final concentrations: ampicillin, 100 μg/ml; chloramphenicol, 25 μg/ml; kanamycin, 50 μg/ml; tetracycline (Tet), 10 μg/ml; rifampin, 100 μg/ml; and 5-bromo-4-chloro-3-indolyl-β-d-galactoside (X-Gal), 40 μg/ml.

**TABLE 1:** Escherichia coli K-12 strains and plasmids used in this study

Strain or plasmid	Relevant genotype or description	Reference(s), source, or construction
Strains		
CAG45114	MG1655 (λ *rpoHP3-lacZ*)	106
ENZ280	Δ(*srlR-recA*)*306*::Tn*10* [mini-F *recA*^+^]	107, 108
FC29	Δ(*lac-proB*)_XIII_ *thi ara* [F′128 *proAB*^+^ *lacI*^q^]	29
FC40	Δ(*lac-proB*)_XIII_ *thi ara* Rif^r^ [F′128 *proAB*^+^ *lacI*^q^ *lacI33-lacZ*]	29
JW3677	BW25113 Δ*recF*::FRT-Kan^r^-FRT	109
JW5437	BW25113 Δ*rpoS*::FRT-Kan^r^-FRT	109
SP874	MC4100 Δ*rpoE*::*cat*	T. Silhavy (Princeton)
SMR820	FC40 *lexA3*(Ind^−^)	24
SMR3856	SMR4562 Lac^+^ day 5	86
SMR4562	Independent construction of FC40	30
SMR5236	SMR4562 *rpoE2072*::Tn*10*dCam	31
SMR5535	SMR4562 Δ*recA*	SMR4562 × P1 (ENZ280)
SMR5833	SMR4562(pKD46)	SMR4562 × pKD46
SMR8842	CAG45114 *rpoE2072*::Tn*10*dCam	CAG45114 × P1 (SMR5236)
SMR10336	SMR4562 Δ*rpoS*::FRT-Kan^r^-FRT	SMR4562 × P1 (JW5437)
SMR10582	SMR4562 *yiaG-yfp* FRT-*cat*-FRT	39
SMR10777	SMR4562 Δ*zie3920.5*::3Chi-Kan-I-SceI cut site	19
SMR10808	FC36 Δ*araBAD*567 Δ*att*λ::P*_BAD_*I-SceI *tet2* FRT	19
SMR10823	FC36 Δ*araBAD567* Δ*att*λ::P*_BAD_*I-SceI *tet2* FRT *rpoE2072*::Tn*10*dCam	SMR10808 × P1 (SMR5236)
SMR10832	FC36 Δ*araBAD567* Δ*att*λ::P*_BAD_*I-SceI *tet2* FRT Δ*rpoS*::FRT	19
SMR10854	FC36 Δ*araBAD567* Δ*att*λ::P*_BAD_*I-SceI *tet2* FRT Δ*zie3920.5*::3Chi-Kan-I-SceI cut site *rpoE2072*::Tn*10*dCam	SMR10823 × P1 (SMR10777)
SMR10862	FC36 Δ*araBAD567* Δ*att*λ::P*_BAD_*I-SceI *tet2* FRT Δ*zie3920.5*::3Chi-Kan-I-SceI cut site Δ*rpoS*::FRT	19
SMR10866	FC36 Δ*araBAD567* Δ*att*λ::P*_BAD_*I-SceI *tet2* FRT Δ*zie3920.5*::3Chi-Kan-I-SceI cut site	19
SMR12566	SMR4562 Δ*rssB*::Tet^r^	39
SMR12661	SMR4562 Δ*rpoS746*::FRT-Kan^r^-FRT *yiaG-yfp* FRT-*cat*-FRT	39
SMR12692	SMR4562 Δ*rssB*::Tet^r^ *yiaG-yfp* FRT-*cat*-FRT	39
SMR12848	SMR4562 *yiaG-yfp* FRT-*cat*-FRT	SMR4562 × P1 (SMR10582)
SMR13014	SMR4562 Δ*rpoS*::FRT	SMR10336 × pCP20
SMR13096	SMR4562 *yiaG-yfp* FRT	39
SMR17962	MG1655 Δ*att*λ::P*_sulA_mCherry* FRT-*cat*-FRT	83
SMR17966	MG1655 Δ*att*λ::P*_sulA_mCherry* FRT *lexA3*(Ind^−^) *malB*::Tn*9*	83
SMR20177	SMR5833 Δ*oxyS*::FRT-*cat*-FRT	This work
SMR20181	SMR5833 Δ*rprA*::FRT-*cat*-FRT	This work
SMR20183	SMR5833 Δ*dsrA*::FRT-*cat*-FRT	This work
SMR20185	SMR5833 Δ*rybB*::FRT-*cat*-FRT	This work
SMR20201	SMR5833 Δ*micF*::FRT-*cat*-FRT	This work
SMR20203	SMR5833 Δ*spf*::FRT-*cat*-FRT	This work
SMR20205	SMR5833 Δ*ryhB*::FRT-*cat*-FRT	This work
SMR20207	SMR5833 Δ*gcvB*::FRT-*cat*-FRT	This work
SMR20219	SMR4562 Δ*oxyS*::FRT-*cat*-FRT	SMR4562 × P1 (SMR20177)
SMR20220	SMR4562 Δ*dsrA*::FRT-*cat*-FRT	SMR4562 × P1 (SMR20181)
SMR20221	SMR4562 Δ*rybB*::FRT-*cat*-FRT	SMR4562 × P1 (SMR20185)
SMR20230	SMR4562 Δ*rprA*::FRT-*cat*-FRT	SMR4562 × P1 (SMR20181)
SMR20232	SMR4562 Δ*micF*::FRT-*cat*-FRT	SMR4562 × P1 (SMR20201)
SMR20234	SMR4562 Δ*spf*::FRT-*cat*-FRT	SMR4562 × P1 (SMR20203)
SMR20236	SMR4562 Δ*ryhB*::FRT-*cat*-FRT	SMR4562 × P1 (SMR20205)
SMR20238	SMR4562 Δ*gcvB*::FRT-*cat*-FRT	SMR4562 × P1 (SMR20207)
SMR20290	SMR4562 Δ*cyaR*::FRT-*cat*-FRT	SMR4562 × P1 (SMR20179)
SMR21332	SMR3856 Δ*gcvB*::FRT-*cat*-FRT	SMR3856 × P1 (SMR20207)
SMR21361	SMR4562 Δ*gcvB*::FRT-*cat*-FRT Δ*rssB*::Tet^r^	SMR12566 × P1 (SMR20207)
SMR21448	SMR4562 Δ*gcvB*::FRT	SMR20238 × pCP20
SMR21450	SMR4562 Δ*micF*::FRT	SMR20232 × pCP20
SMR21467	SMR4562 Δ*rybB*::FRT	SMR20221 × pCP20
SMR21471	SMR4562 Δ*gcvB*::FRT *yiaG-yfp* FRT-*cat*-FRT	SMR21448 × P1 (SMR12848)
SMR21553	SMR21641 Δ*gcvB*::FRT-*cat*-FRT	SMR21641 × P1 (SMR20207)
SMR21633	SMR10866 Δ*gcvB*::FRT-*cat*-FRT	SMR10866 × P1 (SMR20207)
SMR21641	SMR4562 Δ*att*λ::P*_sulA_mCherry* FRT-*cat*-FRT	SMR4562 × P1 (SMR17962)
SMR21725	SMR4562 Δ*rpoS*::FRT Δ*att*λ::P*_sulA_mCherry* FRT-*cat*-FRT	SMR13014 × P1 (SMR17962)
SMR21728	SMR4562 Δ*att*λ::P*_sulA_mCherry* FRT-*cat*-FRT Δ*recF*::FRT-Kan^r^-FRT	SMR21641 × P1 (JW3677)
SMR21909	SMR4562 Δ*rssB*::FRT Δ*gcvB*::FRT	SMR21361 × pCP20
SMR21933	SMR21553 Δ*rssB*::FRT	SMR21909 × P1 (SMR17962)
SMR21934	SMR4562 Δ*gcvB*::FRT Δ*rssB*::Tet^r^ *yiaG-yfp* FRT-*cat*-FRT	SMR21909 × P1 (SMR12848)
SMR21996	SMR4562 Δ*gcvB*::FRT *rpoE2072*::Tn*10*dCam	SMR21448 × P1 (SMR5296)
SMR21998	SMR4562 Δ*gcvB*::FRT *yiaG-yfp* FRT	SMR21471 × pCP20
SMR22047	SMR4562 Δ*gcvB*::FRT *rpoE2072*::Tn*10*dCam *yiaG-yfp* FRT	SMR21998 × P1 (SMR5236)
SMR22064	SMR4562 *yiaG-yfp* FRT *rpoE2072*::Tn*10*dCam	SMR13096 × P1 (SMR5236)
SMR22066	SMR10866 Δ*gcvB*::FRT	SMR21633 × pCP20
SMR22074	SMR21633 *rpoE2072*::Tn*10*dCam	SMR22066 × P1 (SMR5236)
SMR22216	CAG45114Δ*gcvB*::FRT-*cat*-FRT	CAG45114 × P1 (SMR20207)
SMR22296	CAG45114Δ*gcvB*::FRT	SMR22216 × pCP20
SMR22310	SMR22296 *rpoE2072*::Tn*10*dCam	SMR22296 × P1 (SMR5296)
SMR22549	SMR4562 Δ*dsrA*::FRT	SMR20220 × pCP20
SMR22551	SMR4562 Δ*rprA*::FRT	SMR20230 × pCP20
SMR22554	SMR4562 Δ*dsrA*::FRT-*cat*-FRT Δ*rprA*::FRT	SMR22551 × P1 (SMR20220)
SMR22556	SMR4562 Δ*cyaR*::FRT	SMR20290 × pCP20
SMR22558	SMR4562 Δ*oxyS*::FRT	SMR20219 × pCP20
SMR22560	SMR4562 Δ*ryhB*::FRT	SMR20236 × pCP20
SMR22562	SMR4562 Δ*spf*::FRT	SMR20234 × pCP20
SMR22936	SMR3856 Δ*rprA*::FRT-*cat*-FRT	SMR3856 × P1 (SMR20181)
SMR22940	SMR10866 Δ*rprA*::FRT-*cat*-FRT	SMR10808 × P1 (SMR20181)
SMR22950	SMR3856 Δ*rprA*::FRT	SMR22936 × pCP20
SMR22954	SMR10866 Δ*rprA*::FRT	SMR22940 × pCP20
SMR22960	SMR3856 Δ*rprA*::FRT Δ*dsrA*::FRT-*cat*-FRT	SMR22950 × P1 (SMR20183)
SMR22964	SMR10866 Δ*rprA*:FRT Δ*dsrA*::FRT-*cat*-FRT	SMR22954 × P1 (SMR20183)
Plasmids		
pCP20	Temperature-inducible yeast Flp recombinase gene controlled by λ*c*I*ts857* in a temperature-sensitive replicon	110
pKD3	Source of FRT-*cat*-FRT	79
pKD46	*ori101 repA101ts* P*_BAD_gam-bet-exo* Amp^r^	79

Each of nine nonpolar deletions of sRNA genes was constructed by recombineering using pKD3 as the PCR template (79). The nucleotides deleted for each new deletion allele are shown in Table S1 in the supplemental material.

### Quantitative Lac mutagenesis assays with spontaneous DSBs.

The Lac assay (29) for stress-inducible MBR measures reversion of an F′-borne *lacI-Z* gene with a +1-bp frameshift allele during starvation stress, and the assay was performed as described previously (23). Viable Lac^−^ starving cells on the lactose-containing plates were measured daily throughout the experiments as described previously (29) and varied less than 2-fold during the days of the experiments reported. Lac^+^ revertant CFU (indel mutants) are counted to day 5. The mutation rates (Lac^+^ CFU per 10^8^ CFU per day) shown are means ± standard errors of the means (SEM) from four separate experiments with four independent cultures for each strain and were calculated as described previously (28) by subtracting the number of colonies counted on day 3 from the number of colonies counted on day 5 and dividing by 2.

### Chromosomal Tet reversion assay with I-SceI-induced DSBs in plasmid-free cells.

The Tet reversion assay of Shee et al. (19, 20, 39) was performed as described previously. A chromosomally encoded arabinose-inducible, glucose-repressible I-SceI endonuclease, produced weakly by leaky expression, cleaves a chromosomal I-SceI cut site (I-site) near a *tet* mutation reporter gene in liquid-starved plasmid-free cells (19). The chromosomal *tet* gene with a +1-bp frameshift allele resides 8.5 kb from the I-site (I-site A, *tet2*) (19). Cells are grown for 12 h to saturation in liquid, starved for 72 further hours, then rescued from starvation, plated on LBH solid medium containing glucose and tetracycline (LBH glucose tetracycline solid medium), and incubated at 37°C to select *tet*^+^ revertant tetracycline-resistant (Tet^r^) colonies, which are counted the following day, as are the total viable CFU assayed on medium without tetracycline. Mutant frequencies are the titers of Tet^r^ mutant CFU per milliliter on LBH glucose tetracycline medium divided by those of total CFU/ml from LBH glucose plates. Data presented are the values (means ± SEM) from 10 independent experiments with three cultures for each strain.

### Flow cytometric assays for σ^S^ and SOS response-regulated promoter activity.

σ^S^ and SOS response activation for σ^S^ activity were quantified by flow cytometry as described previously (39) for σ^S^. The method of Pennington and Rosenberg (82) as modified by Nehring et al. (83) was used for SOS. The chromosomal *yiaG-yfp* σ^S^ response reporter gene (39) and Δ*att*λ::P_*sulA*_*mCherry* SOS reporter gene (83) were used in separate cells. Strains were grown at 37°C for 48 h with aeration in liquid M9 medium containing vitamin B_1_ and glycerol (liquid M9 B1 glycerol). σ^S^ and SOS response-dependent promoter activity was quantified in two ways. First, for the SOS response, in which only a small subpopulation of growing cells is induced spontaneously relative to negative-control, SOS-off mutant cells (82), we set gates by the method of Pennington and Rosenberg (82). Gates were calibrated using negative-control SOS-off *lexA*(Ind^−^) cells, and SOS response “on” was scored as the fluorescence intensity shown by the most fluorescent 1% of events observed in wild-type cultures. Cells that fell below this gate (less fluorescence) were scored as negative. The values (percent positive cells; means ± SEM) from five independent SOS activity experiments with three independent cultures for each strain are given. Second, for both SOS and σ^S^ responses, we report the mean fluorescence intensity per cell, a measure more useful for responses and mutants in which cells display a unimodal distribution of fluorescence intensities, and a majority or all of the cells in the population of mutants examined are shifted relative to the wild-type strain or the negative-control strains.

### Western blot analyses of σ^S^ and σ^E^ protein levels.

Western blot analyses for quantification of σ^S^ and σ^E^ protein levels in stationary cultures were performed by the methods of Galhardo et al. (33) and Gibson et al. (31), respectively. The optical density at 600 nm (OD_600_) was taken of 5-ml samples from 48-h cultures grown in M9 B1 glycerol, and the concentrations were adjusted to standardize the different strains. The cells were pelleted and resuspended in 1 ml of lysis buffer/loading sample (62.5 mm Tris [pH 6.8], 25% glycerol, 2% SDS, 0.01% bromophenol blue, 0.5% β-mercaptoethanol) and boiled. Fifteen-microliter portions of each sample were electrophoresed on 13% SDS–polyacrylamide gels, and the proteins were transferred to Hybond-LFP polyvinylidene difluoride (PVDF) membranes (Amersham Biosciences). The membranes were blocked with 2% blocking buffer and probed with 1:700 dilution of polyclonal mouse anti-σ^S^ antibody (Neoclone) (84) or 1:5,000 dilution of polyclonal rabbit anti-σ^E^ antibody (85) (gift of Carol Gross, University of California at San Francisco [UCSF]). Goat anti-mouse and anti-rabbit secondary antibodies conjugated to Cy5 fluorescent dye (Amersham Biosciences) were used at a 1:5,000 dilution to detect σ^S^ and σ^E^ proteins, respectively. Fluorescence was assessed on a Typhoon scanner with a photomultiplier voltage (PMT) of 500 and a 670-nm bandpass (670BP) 30Cy5 emission filter, and the bands were quantified using ImageJ software (NIH). Quantifications from four separate Western blots for σ^S^ and σ^E^ are reported, each with band intensities normalized to isogenic wild-type control strain SMR4562 and means ± 1 SEM are shown.

### Reconstruction experiments.

Reconstruction experiments were used to demonstrate that Lac^+^ Δ*gcvB* and Lac^+^ Δ*dsrA* Δ*rprA* cells form colonies normally under selective assay conditions in the presence of neighbor cells, using assays described previously (86) as reviewed in reference 87, such that their defect in producing Lac^+^ mutant colonies reflects reduced mutagenesis, not impaired colony formation. We quantified the timing of colony appearance and fraction of known numbers of Δ*gcvB* and Δ*dsrA* Δ*rprA* CFU that formed colonies under precise reconstructions of experimental conditions and compared these with those of isogenic nonmutant strains.

### UV light sensitivity assays.

Saturated liquid M9 B1 glycerol cultures were starved as in the Lac mutagenesis assays described above, diluted, plated at various concentrations onto LBH solid medium, and exposed to various doses of UVC light in a Stratalinker (Artisan Technology Group). CFU titers were quantified and graphed. Data are normalized to viable-cell titers with no UVC irradiation. *lexA*(Ind^−^) mutant cells, which produce an uncleavable LexA transcriptional repressor of the SOS genes, and so are SOS response defective, were used as a positive control for SOS response deficiency. Δ*recA* cells have stronger UV sensitivity and were also used as a positive control.

### Semiquantitative SDS-EDTA sensitivity assay for σ^E^ response deficiency.

σ^E^ response-defective cells are sensitive to SDS-EDTA, which disrupts the membrane (88). Strains grown to saturation and starved, per Lac mutagenesis experiments, were diluted, and 10-μl spots containing ∼30 and ∼300 CFU deposited onto solid M9 B1 glycerol medium with and without 0.01% SDS and 0.25 mM EDTA, incubated for 48 h at 30°C (the permissive temperature for a Δ*rpoE* control strain [89]) and scored. We used control isogenic cells carrying the *rpoE2072*::Tn*10*dCam separation-of-function allele, which confers σ^E^ response deficiency but maintains the essential function of σ^E^ (31), and are SDS-EDTA sensitive (31). Although *rpoE* is an essential gene (89), the Δ*rpoE* mutant is viable because of acquisition of compensatory extragenic “suppressor” mutations that permit viability (90).

### Catalase colony assays for σ^S^ response activity.

Catalase colony assays for σ^S^ response activity were performed as described previously (91). Wild-type control, isogenic Δ*gcvB*, and Δ*rpoS* strains were grown into colonies on M9 B1 glycerol medium for 48 h at 37°C. Three microliters of 30% hydrogen peroxide was dropped onto each colony, and the time elapsed before bubbles appeared was measured. Six colonies were tested in four independent experiments, and the times to bubbling (means ± SEM) (in seconds) were reported. *P* values compared with the values for wild-type colonies were determined using two-tailed Student's *t* test.

### β-Galactosidase assay for σ^E^ activity.

Because the *rpoH* P3 promoter is σ^E^ dependent, the *rpoHP3-lacZ* fusion gene is a reporter for σ^E^-dependent transcription (92), which is measured as β-galactosidase activity in liquid cultures. β-Galactosidase assays of saturated M9 B1 glycerol cultures were performed as described previously (31). The values (means ± SEM) from three experiments and four independent cultures for each strain are reported.

### Acid sensitivity assays.

Acid sensitivity assays were performed as described previously (65). Saturated overnight cultures of wild-type control and isogenic Δ*gcvB*, *rpoE2072*::Tn*10*dCam (*rpoE*::Tn), and Δ*gcvB rpoE*::Tn cells were diluted 1:50 in LB medium and grown at 37°C with aeration for 5 h. Acid challenge was performed by adding 3 volumes of acidified LB medium (pH 1.9) to cultures, resulting in a final pH of 2.0. Cells were grown in acidified liquid culture for 30 min. The challenge was interrupted with the addition of 3 volumes of alkalinized LB medium (pH 9.3), resulting in a final pH of 7.0. The optical density at 600 nm was taken after 3 h of recovery. The values (means ± SEM) for three experiments containing three independent cultures per strain are reported.

## RESULTS

### MBR deficiency caused by deletion of Hfq client genes *gcvB* and *dsrA* or *rprA*.

**FIG 1:**
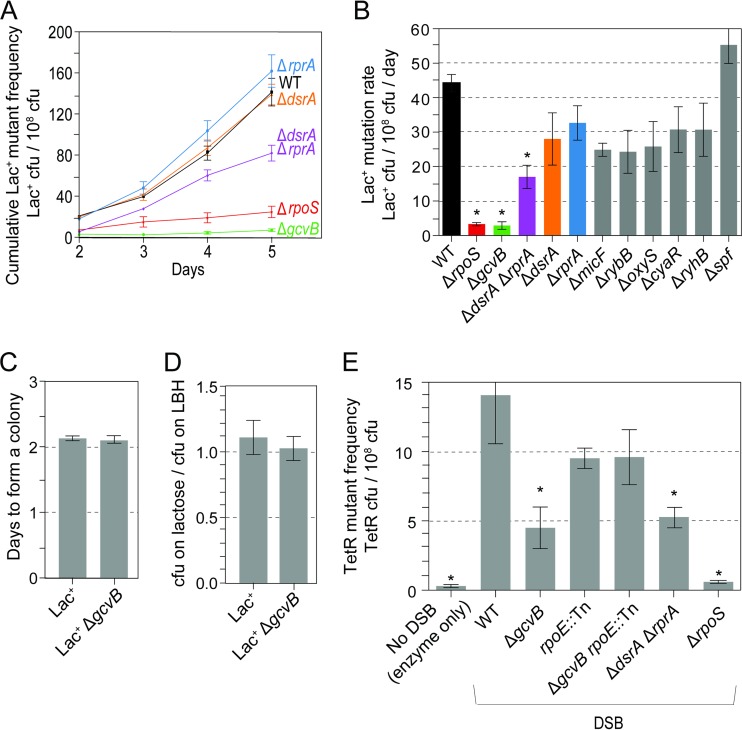
sRNA GcvB promotes mutagenic break repair in chromosomal and F′-based MBR assays. (A) Lac MBR assay. Lac^+^ CFU are revertants of a conjugative-plasmid-borne *lac* frameshift allele during starvation on solid medium. The results of a representative experiment are shown. (B) Quantification of Lac^+^ MBR mutation rates as described in Materials and Methods. The values are means ± standard errors of the means (SEM) (error bars) from three experiments for each strain. Asterisks indicate values that are significantly different from the value for the isogenic wild-type (WT) control strain (*P* = 0.0002 for the Δ*rpoS* strain, *P* = 0.0004 for the Δ*gcvB* strain, and *P* = 0.01 for the Δ*dsrA* Δ*rprA* strain; two-tailed Student's *t* test used in all comparisons here). From left to right, isogenic strains are SMR4562, SMR10336, SMR21448, SMR22554, SMR22549, SMR22551, SMR21450, SMR21467, SMR22558, SMR22556, SMR22560, and SMR22562. (C and D) Reconstruction experiments show that, when constructed, Δ*gcvB* mutant Lac^+^ cells are proficient at colony formation (Materials and Methods). (C) Normal speed of colony formation by Δ*gcvB* cells under MBR assay conditions. (D) Similar efficiencies of colony formation under selective conditions, compared with CFU on rich (LBH) medium without neighbor cells. (E) Chromosomal Tet MBR assay in plasmid-free cells. Δ*gcvB* cells display a MBR defect that is relieved by the *rpoE*::Tn separation-of-function mutation, which blocks the membrane stress response (31). Asterisks indicate values that are significantly different from the value for the isogenic wild-type (WT) control strain (*P* = 0.04 for the Δ*gcvB* strain, *P* = 0.04 for the Δ*dsrA* Δ*rprA* strain, *P* = 0.001 for the Δ*rpoS* strain, and *P* = 0.001 for “No DSB” [I-SceI enzyme present with no cut site]). Values are means ± SEM from seven experiments with positive controls. From left to right, the strains are SMR10808, SMR10866, SMR21633, SMR10854, SMR22074, SMR22964, and SMR10862. TetR, tetracycline resistant.

We deleted nine genes that encode sRNA clients of Hfq (see Table S1 in the supplemental material), genes that are not embedded in another gene and that showed expression patterns potentially relevant to starvation stress (59). We assayed the deletion mutants for MBR proficiency/deficiency using the Lac MBR assay (29). The Lac assay quantifies reversion via MBR of a conjugative-plasmid-borne *lac* gene with a +1-bp frameshift allele during prolonged starvation for days on minimal lactose solid medium (1–3, 6, 7). Colonies visible on day 2 are roughly 50% preexisting generation-dependent Lac^+^ reversion mutants, and colonies from day 3 onward are DSB-, DinB/Pol IV-, SOS-, σ^S^-dependent, MBR-generated revertants. Δ*hfq* cells show a strong 16-fold ± 2-fold deficiency in MBR in the Lac assay (39). We found that Δ*gcvB* cells showed a significant 12-fold ± 2-fold defect in Lac^+^ MBR revertant accumulation (Fig. 1A and B, mean ± SEM mutation rate compared with the WT), a defect smaller than that of the Δ*hfq* mutant. The double mutant Δ*gcvB Δhfq* was inviable and could not be tested. In addition, the Δ*dsrA ΔrprA* double mutant showed a smaller but significant 2-fold ± 0.4-fold reduction in the accumulation of Lac^+^ revertants (Fig. 1A and B). Because neither the Δ*dsrA* nor Δ*rprA* single mutant showed reduction, the data imply that either DsrA or RprA can function in MBR (they are redundant functions), such that loss of neither sRNA singly reduces accumulation of revertants. Both sRNAs promote translation of σ^S^ (54). Here, we follow up the role of sRNA GcvB.

### GcvB and RprA or DsrA are required for mutagenesis, not mutant colony formation.

We show that the failure of Δ*gcvB* cells to produce Lac^+^ revertant colonies (Fig. 1A and B) is not merely the inability of Lac^+^ revertants carrying a Δ*gcvB* mutation to form colonies under experimental conditions. We performed reconstruction experiments in which a functional Lac^+^ allele is moved into Δ*gcvB* cells, and their efficiency and speed of colony formation under precise reconstructions of experimental conditions are measured (∼100 Lac^+^ Δ*gcvB* cells mixed with ∼10^9^ Δ*lac* nonrevertible neighbor cells on selective plates). The data show that Δ*gcvB* cells form colonies normally and do not have decreased viability or growth rate under experimental conditions (Fig. 1C and D). Similar reconstruction experiments showed that Δ*dsrA* Δ*rprA* mutant cells also do not have decreased ability to form colonies under experimental conditions (see Fig. S1 in the supplemental material). We conclude that GcvB is required for mutagenesis, not outgrowth of mutant cells into colonies, as are DsrA or RprA.

### GcvB and either DsrA or RprA are required for MBR in the chromosomal Tet assay.

We confirmed the MBR deficiency of Δ*gcvB* and Δ*dsrA* Δ*rprA* cells using the chromosomal Tet MBR assay (19) in which a chromosomal *tet* gene in plasmid-free cells reverts by indel mutation during prolonged starvation in liquid and then the cells are rescued from starvation and selected for tetracycline-resistant (Tet^r^) mutant CFU. A chromosomally encoded I-SceI endonuclease is weakly induced and cleaves an I-SceI cut site near the *tet* gene, provoking repair, presumably with a sister chromosome (present in ∼40% of stationary-phase E. coli [93]). The *tet* reversions that result are dependent on DSBs, DSB repair protein, SOS, DinB, and σ^S^ (19), all as observed in the Lac assay (18). We found that GcvB and either DsrA or RprA promoted a significant 69% ± 11% and 43% ± 9% of MBR, respectively, in the chromosomal Tet assay (Fig. 1E). We conclude that GcvB promotes much of stress-inducible MBR in E. coli, generally.

### GcvB promotes MBR other than or in addition to by promoting SOS, DSB repair, the σ^E^ response, or spontaneous DNA breakage.

**FIG 2:**
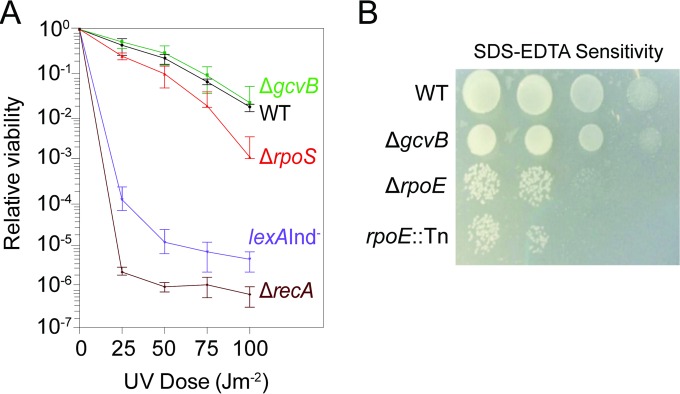
Δ*gcvB* cells show normal recombinational DNA repair and activation of the SOS and σ^E^ stress responses. (A) UV sensitivity assay. Values are means ± SEM from three experiments with two cultures for each experiment. The UV resistance of Δ*gcvB* cells indicates homologous recombinational (HR) repair and SOS response proficiency. Δ*recA* HR- and SOS-defective cells and SOS-uninducible *lexA*(Ind^−^) cells are UV sensitive (*P* < 0.02 compared with the value for the WT; Student's two-tailed paired *t* test for each UV dose). The mild UV sensitivity of *rpoS*-null cells was observed previously (105). The lack of UV sensitivity of Δ*gcvB* cells indicates that their σ^S^ response impairment (Fig. 3) is not as severe as in σ^S^-null cells (Fig. 3B). From top to bottom, the strains are SMR20238, SMR4562, SMR10336, SMR820, and SMR5535. (B) Δ*gcvB* cells are SDS-EDTA resistant, indicating a functional σ^E^ response. Shown is a representative image of cultures spotted onto solid medium containing membrane-disrupting detergent (SDS) and EDTA at ∼300 CFU per spot (left two spots) and ∼30 CFU per spot (right two spots). SDS and EDTA retard growth of σ^E^-response-defective Δ*rpoE* (88) and *rpoE*::Tn*10*dCam (31) mutant cells. Although *rpoE* is an essential gene (89), the Δ*rpoE* mutant is viable because of acquisition of compensatory extragenic “suppressor” mutations that permit viability (90). From top to bottom, the strains are SMR4562, SMR20238, MC4100, and SMR5236.

We tested Δ*gcvB* cells for possible defects in several known components of MBR reactions. Cells defective for homologous recombinational (HR) DSB repair or the SOS response both show sensitivity to UV light (94, 95) [Fig. 2A, *lexA*(Ind^−^) and Δ*recA* positive-control strains]. We found that Δ*gcvB* cells were as UV resistant as isogenic *gcvB*^*+*^ cells (“wild-type” [WT] cells in Fig. 2A), indicating that they have neither SOS nor HR defects. Two lines of evidence show that a defective σ^E^ membrane stress response does not underlie the MBR deficiency of Δ*gcvB* cells. First, cells with defects in the σ^E^ membrane stress response show sensitivity to SDS-EDTA, which disrupts the cell membrane (88). We found that Δ*gcvB* cells were as resistant to SDS-EDTA as the wild-type isogenic control (Fig. 2B). Second, σ^E^ promotes MBR in the Lac assay by promoting spontaneous DNA breakage (31); thus, σ^E^ is not required in the Tet assay in which DSBs are provided by I-SceI endonuclease (19, 39). Our finding that GcvB also promotes MBR in the Tet assay (Fig. 1E) indicates that GcvB promotes MBR other than or in addition to by allowing a σ^E^ response and other than or in addition to by promoting spontaneous DNA breakage. Because Δ*gcvB* cells showed a greater reduction of MBR in the Lac assay than in the Tet assay, it remains possible that GcvB plays two roles: one that affects spontaneous DNA breakage and another DSB-/σ^E^-independent role.

### Decreased σ^S^ response but normal σ^S^ protein levels in cells that lack GcvB.

**FIG 3:**
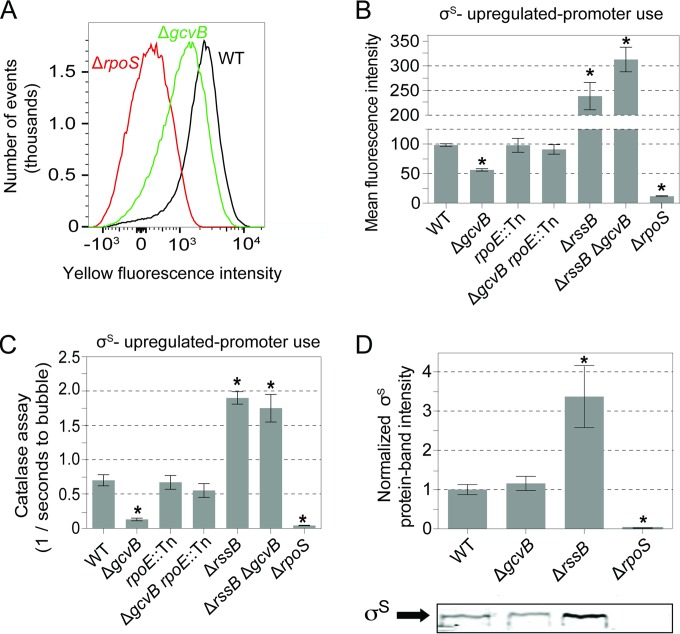
Reduced σ^S^-upregulated transcription, but not σ^S^ protein levels, in stationary-phase Δ*gcvB* cells and its dependence on the σ^E^ response. In all experiments, measurements and assays are from stationary-phase cells, grown under experimental conditions as for mutagenesis experiments. (A) Reduced σ^S^-regulated promoter activity in Δ*gcvB* cells. A flow cytometric fluorescence assay of stationary-phase starved cultures shows σ^S^-dependent yellow fluorescence (in arbitrary fluorescence units) from the *yiaG-yfp* σ^S^ response reporter gene (39). The results of a representative experiment are shown. (B) Quantification of mean fluorescence intensities per cell from five independent experiments. Fluorescence intensity is shown in arbitrary fluorescence units. Values are means ± SEM. The *rpoE*::Tn σ^E^-response-defective mutation (31) restored σ^S^ response activity to Δ*gcvB* cells, indicating that the σ^S^ response reduction in Δ*gcvB* cells is σ^E^ response dependent. Asterisks indicate values that are significantly different from the value for the WT strain (*P* = 1 × 10^−6^ for the Δ*gcvB* strain, *P* = 4 × 10^−8^ for the Δ*rssB* strain, *P* = 9 × 10^−8^ for the Δ*rssB* Δ*gcvB* strain, and *P* = 2 × 10^−10^ for the Δ*rpoS* strain) by Student's two-tailed *t* test. The value for the Δ*gcvB rpoE*::Tn double mutant is significantly different from the value for the Δ*gcvB* single mutant (*P* = 4 × 10^−3^). There is no significant difference between the values for the Δ*rssB* mutant and Δ*rssB* Δ*gcvB* mutant (*P* = 0.56 by Student's two-tailed *t* test). From left to right, the strains are SMR10582, SMR21471, SMR22064, SMR22047, SMR12692, SMR21934, and SMR12661. (C) σ^E^-response-dependent reduction of σ^S^ response activity in Δ*gcvB* cells by the catalase colony assay (Materials and Methods). Asterisks indicate values that are significantly different from the value for the WT strain (*P* = 8 × 10^−5^ for the Δ*gcvB* strain, *P* = 4 × 10^−13^ for the Δ*rpoS* strain, *P* = 2 × 10^−4^ for the Δ*rssB* strain, and *P* = 0.01 for the Δ*rssB* Δ*gcvB* strain. Values are means ± SEM from four experiments with five colonies per experiment. The strains are the same strains used in panel B. (D) Western blot analyses show σ^S^ protein levels in stationary phase unaffected by the Δ*gcvB* mutation. (Top) Results from three quantified immunoblots normalized to the WT value. Values are means ± SEM. Asterisks indicate values that are significantly different from the value for the WT strain (*P* = 1 × 10^−3^ for the Δ*rpoS* strain and *P* = 7 × 10^−4^ for the Δ*rssB* strain). (Bottom) Representative immunoblot. From left to right, the strains are SMR4562, SMR20238, SMR12566, and SMR10336.

We found that Δ*gcvB* cells display reduced activity of σ^S^-upregulated promoters in two assays (Fig. 3). First, cells defective for the σ^S^ response have decreased *katE* transcription and thus decreased catalase activity and a defect in metabolizing hydrogen peroxide (H_2_O_2_) (96). When a drop of hydrogen peroxide is placed on an E. coli colony, H_2_O_2_ is metabolized to H_2_O and O_2_, and bubbles appear on the colony. The rapidity of onset of bubbling indicates σ^S^-upregulated promoter activity (96). For wild-type and Δ*rpoS* strains, bubbles appeared after 2 ± 0.3 and 22 ± 1 s, respectively (Fig. 3C, plotted as 1/time to bubbling). Δ*gcvB* cells take 11 ± 1 s (mean ± SEM) to produce bubbles, which is significantly different from the wild-type cells and not significantly different from the σ^S^-null Δ*rpoS* strain data (Fig. 3C).

Second, we measured activity of the σ^S^-upregulated *yiaG* promoter using flow cytometry of cells carrying a chromosomal *yiaG-yfp* reporter gene (39), which exploits the σ^S^ specificity of the *yiaG* promoter (Fig. 3A). We found that Δ*gcvB* cells showed a significant 1.6-fold ± 0.08-fold decrease in mean yellow fluorescence intensity (per cell) compared with the WT control (Fig. 3B). The decreased production of yellow fluorescent protein (YFP) from P_*yiaG*_ is not as great as in σ^S^-null Δ*rpoS* cells (Fig. 3A and B), implying that σ^S^-regulated promoter activity is reduced but not abolished in Δ*gcvB* cells. The reductions in expression of σ^S^-upregulated genes in both assays were reversed by artificial upregulation of σ^S^ via deletion of *rssB* (Fig. 3B and C). RssB is a protein chaperone that brings σ^S^ to the ClpXP protease for degradation (97), such that its removal causes artificially high σ^S^ levels (97).

Although σ^S^-upregulated promoter activity is reduced in Δ*gcvB* cells, we found that σ^S^ protein levels are not detectably reduced relative to isogenic *gcvB*^*+*^ (“wild-type”) cells. A representative Western blot is shown in Fig. 3D. Quantification from multiple Western blots shows that the relative levels of σ^S^ protein in Δ*gcvB* cells compared to wild-type cells are 1.1 ± 0.1 and 1.0 ± 0.1, respectively (Fig. 3D, wild-type normalized to 1.0, mean ± SEM from four experiments, the SEM on the WT is normalized proportionally to 1 to show the day-to-day variability in measurement). These data imply that GcvB does not regulate σ^S^ production or stability in stationary cells. We conclude that σ^S^ is present in Δ*gcvB* cells but that σ^S^ -upregulated promoter activity is decreased.

### Mutagenesis and high spontaneous SOS induction in Δ*gcvB* cells are relieved by artificial upregulation of σ^S^.

**FIG 4:**
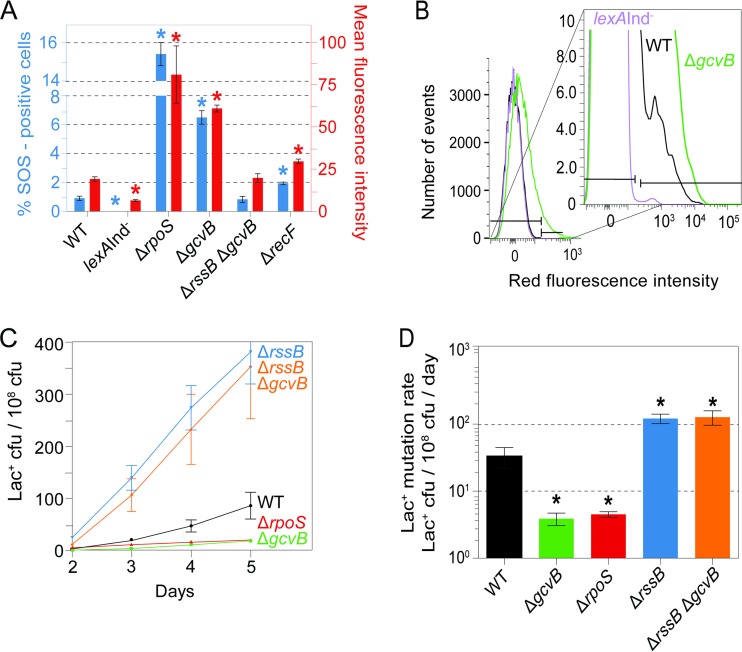
Artificial upregulation of σ^S^ substitutes for GcvB in mutagenesis and suppression of spontaneous SOS induction. (A) Increased spontaneous SOS response in Δ*rpoS* or Δ*gcvB* cells and its reversibility in Δ*gcvB* cells by the Δ*rssB* mutation, which promotes σ^S^ stability. SOS activity was measured by the method of Pennington and Rosenberg (82) as modified by Nehring et al. (83) by flow cytometry of strains with a chromosomal SOS-upregulated fluorescence reporter transgene, Δ*att*λ::P_*sulA*_*mCherry*. This assay quantifies single cells with spontaneous DNA damage that triggers the SOS response (not spurious promoter firing, shown in reference 82). On the left-hand *y* axis, the percentage of SOS-positive cells quantifies the fraction of cells in a subpopulation with higher fluorescence than the main population. Gates (horizontal brackets shown in panel B) are set per Materials and Methods for the bimodal distribution in wild-type cells, also shown in panel B. The right-hand *y* axis shows the mean fluorescence intensity (in arbitrary fluorescence units per cell) of all single cells assayed. The mean fluorescence intensity reports on shifts that affect most of the cells in the population, as seen in Δ*gcvB* cells (shown also in panel B). The data imply that the σ^S^ response prevents some spontaneous DNA damage. Asterisks indicate values that are significantly different from the value for the WT strain [*P* = 2 × 10^−6^ for the *lexA*(Ind^−^) strain, *P* = 4 × 10^−10^ for the Δ*gcvB* strain, *P* = 5 × 10^−4^ for the Δ*rpoS* strain, and *P* = 5 × 10^−6^ for the Δ*recF* strain]. Values are means ± SEM from four experiments. From left to right, the strains are SMR21641, SMR17966, SMR21725, SMR21553, SMR21933, and SMR21728. (B) Representative flow cytometry analysis of spontaneous SOS induction showing the small cell subpopulation with spontaneous SOS-activating DNA damage, as described previously (82), in arbitrary fluorescence units. (C) The Δ*gcvB* MBR-deficient phenotype is suppressed by Δ*rssB*, a mutation that increases σ^S^ protein levels by reducing σ^S^ proteolytic degradation (97), and increases MBR as shown here and shown previously (39, 98). The results of a representative experiment are shown. (D) Quantification of mutation rates from three experiments. Values are means ± SEM. Asterisks indicate values that are significantly different from the value for the WT strain (*P* = 0.05 for the Δ*gcvB* strain, *P* = 0.05 for the Δ*rpoS* strain, *P* = 0.006 for the Δ*rssB* strain, and *P* = 0.04 for the Δ*gcvB* Δ*rssB* strain) by Student's two-tailed *t* test. From left to right, the strains are SMR4562, SMR20238, SMR10336, SMR12566, and SMR21361.

We observed previously that Δ*rpoS* cells, which lack a functional σ^S^ response, display increased spontaneous induction of the SOS response (98) (Fig. 4A) for unknown reasons. Perhaps damaged cellular components and/or high levels of reactive oxygen species, normally reduced by the σ^S^ response, cause DNA damage. We also found increased spontaneous SOS induction in Δ*gcvB* cells (Fig. 4A and B). We found that both the mutagenesis defect and the high spontaneous SOS response in Δ*gcvB* cells were reversed by deletion of *rssB*, which upregulates σ^S^ by reducing ClpXP-mediated proteolytic degradation of σ^S^ (97). Deletion of *rssB* increases the general stress response (97), and it also increases MBR (39). *rssB* deletion fully restored mutagenesis to Δ*gcvB* cells, elevated mutagenesis to the greater-than-wild-type levels seen in Δ*rssB* cells (Fig. 4C and D; see also Table S2 in the supplemental material), and ameliorated the Δ*gcvB* high-SOS phenotype (Fig. 4A). The data indicate that GcvB promotes MBR by allowing a robust σ^S^ response, such that in Δ*gcvB* cells, MBR is reduced via σ^S^ response deficiency.

### Blocking the σ^E^ membrane stress response restores mutagenesis and σ^S^ response activity to Δ*gcvB* cells.

We tested the hypothesis that reduced transcription of σ^S^-upregulated genes and reduced MBR in Δ*gcvB* cells (Fig. 1, 3, and 4) might result from hyperinduction of the σ^E^ membrane stress response. The σ^E^ response promotes MBR by promoting spontaneous DNA DSBs by as yet unknown means (31). Although σ^E^ is an essential protein, encoded by *rpoE*, we previously isolated a separation-of-function *rpoE* mutation, *rpoE2072*::Tn*10*dCam (*rpoE*::Tn), that retains the σ^E^ essential function but is incapable of mounting a σ^E^ stress response (31). Cells carrying this special *rpoE*::Tn mutation show a ≥10-fold reduction in spontaneous MBR but no reduction if DSBs are supplied by I-SceI double-strand endonuclease (31). Because the Tet MBR assay measures mutagenesis activated by I-SceI cleavage at a nearby *tet* gene (19), MBR in the Tet assay is σ^E^ independent. We used the Tet MBR assay to test whether a σ^E^ response interferes with σ^S^-dependent promoter activity and thus MBR. We found that the *rpoE*::Tn mutation restored normal levels of MBR to Δ*gcvB* cells (Fig. 1E, Δ*gcvB rpoE*::Tn compared with WT) and normal σ^S^-dependent-promoter activity measured with flow cytometric and colony/catalase assays (Fig. 3B and C, Δ*gcvB rpoE*::Tn compared with WT). We conclude that the σ^E^ role in the membrane stress response underlies the defects in σ^S^-dependent promoter activity and MBR in Δ*gcvB* cells. This might result from hyperinduction of the σ^E^ response in Δ*gcvB* cells, in which excessive σ^E^ molecules titrate RNA polymerase (RNAP), decreasing normal levels of σ^S^-RNAP enzyme in favor of the σ^E^-RNAP enzyme, thus reducing the σ^S^ response. Whereas *rpoE*::Tn increased σ^S^-dependent promoter activity and MBR in Δ*gcvB* cells (Fig. 1E and 3B and C), *rpoE*::Tn did not increase σ^S^ protein levels in Δ*gcvB* cells (see Fig. S2 in the supplemental material), supporting the σ^E^/σ^S^ competition hypothesis.

### σ^E^ activity and protein levels are increased in Δ*gcvB* cells.

**FIG 5:**
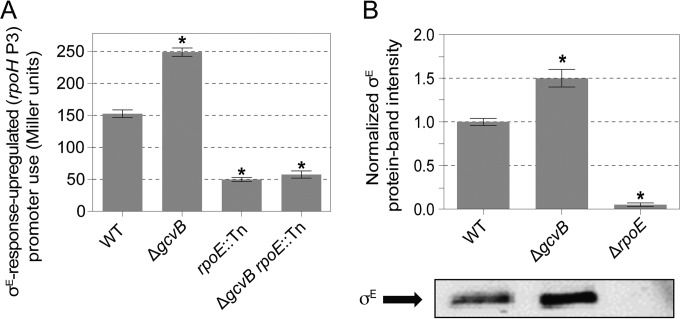
Hyperactivation of the σ^E^ membrane stress response in cells that lack sRNA GcvB. (A) Increased activation of the σ^E^-dependent *rpoH* P3 promoter in stationary-phase Δ*gcvB* cells measured as β-galactosidase activity from the *rpoHP3-lacZ* fusion gene. Values are means ± SEM from three experiments. Asterisks indicate values that are significantly different from the value for the WT strain (*P* = 4 × 10^−3^ for the Δ*gcvB* strain, *P* = 2 × 10^−3^ for the *rpoE*::Tn strain, and *P* = 3 × 10^−3^ for the Δ*gcvB rpoE*::Tn strain). From left to right, the strains are SMR8841, SMR22216, SMR8842, and SMR22310. (B) σ^E^ protein levels are increased in cells that lack GcvB. (Top) Quantified Western immunoblots normalized to WT bands. Values are means ± SEM from three experiments. Asterisks indicate values that are significantly different from the value for the WT strain (*P* = 7 × 10^−4^ for the Δ*gcvB* strain and *P* = 5 × 10^−6^ for the Δ*rpoE* strain). (Bottom) Representative immunoblot. From left to right, the strains are SMR4562, SMR20238, and MC4100.

We measured σ^E^ activity using the *rpoHP3-lacZ* promoter fusion reporter gene (92), which reports on σ^E^ stress response-dependent transcription as β-galactosidase activity (92). We found a significant 1.6-fold ± 0.04-fold increase in σ^E^-dependent β-galactosidase activity in Δ*gcvB* cells relative to their isogenic *gcvB*^*+*^ parent cells (Fig. 5A). We also found that σ^E^ protein levels were increased 1.5-fold ± 0.1-fold in Δ*gcvB* cells (mean ± SEM for four Western blots [a representative blot shown in Fig. 5B]). The data imply that normally, GcvB plays a role that suppresses the σ^E^ stress response. GcvB might simply function in some way that promotes membrane integrity by, for example, maintaining proper levels of membrane proteins such that upon its removal, the σ^E^ response is induced.

### The σ^E^ response is required for acid resistance.

**FIG 6:**
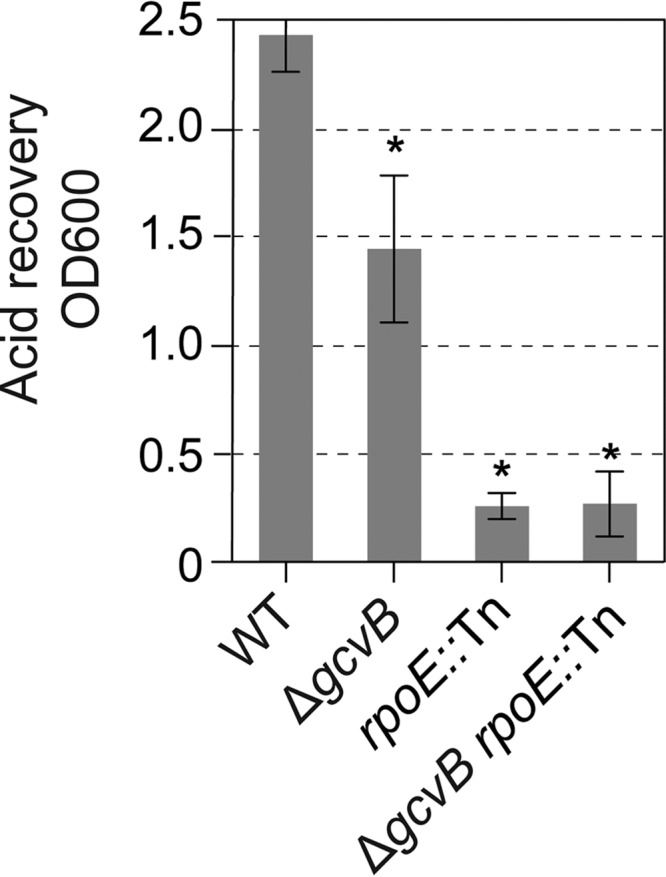
The σ^E^ response is required for acid resistance. The *rpoE2072*::Tn mutation, which ablates the σ^E^ response without affecting the σ^E^ essential function (31), caused strong acid sensitivity, indicating that the σ^E^ response is required for acid resistance. Because Δ*gcvB* cells display a hyper-σ^E^ response (Fig. 5) and *rpoE*::Tn cells have no σ^E^ response (31), the data suggest that both too much and too little σ^E^ response activity may result in acid sensitivity and that the σ^E^ response must occur at just the right level for resistance. The OD_600_ of cultures after 3-h recovery from a 30-min acid challenge was measured. Values are means ± SEM from three experiments with three independent cultures for each strain. Asterisks indicate values that are significantly different from the value for the wild-type (WT) control strain (*P* = 0.01 for the Δ*gcvB* strain, *P* = 5 × 10^−8^ for the *rpoE*::Tn strain, and *P* = 3 × 10^−9^ for the Δ*gcvB rpoE*::Tn strain) by Student's two-tailed paired *t* test. From left to right, isogenic strains are SMR4562, SMR20238, SMR5236, and SMR21996.

We attempted to test whether the acid sensitivity of the E. coli Δ*gcvB* mutant (65) might, like MBR, result from hyperinduction of the σ^E^ response. Surprisingly, we found that cells that carry the *rpoE2072*::Tn allele, which blocks the σ^E^ stress response without impairing the σ^E^ essential function (31), were also acid sensitive, and more acid sensitive than Δ*gcvB* cells were (Fig. 6). We conclude that a functional σ^E^ response is required for acid resistance. This is not incompatible with the possibility that the acid sensitivity of the E. coli Δ*gcvB* cells (65) results from a hyperinduced σ^E^ response, implying that both too much of a σ^E^ response and too little result in acid sensitivity. Further experiments would be needed to establish that specific mechanism.

## DISCUSSION

We found that GcvB, an sRNA client of Hfq, promotes mutagenic break repair (MBR) during starvation stress in E. coli in two different MBR assays (Fig. 1) and presented evidence that it does so by allowing a robust σ^S^ general/starvation stress response, apparently by suppressing the σ^E^ membrane stress response. First, MBR proficiency was restored to Δ*gcvB* cells by artificial upregulation of σ^S^ using an *rssB* mutation, which blocks σ^S^ protein degradation (Fig. 4C and D), implying that the Δ*gcvB* MBR defect is caused by failure to mount a robust σ^S^ response. Moreover, we found that (i) cells that lack GcvB showed decreased σ^S^-regulated gene expression in two assays (Fig. 3A to C) and that (ii) the σ^S^-dependent reduction in gene expression was also reversible by σ^S^ upregulation (Fig. 3B and C) but that (iii) σ^S^ protein levels were not reduced in Δ*gcvB* cells (Fig. 3D).

Second, blocking the σ^E^ membrane stress response, but not the σ^E^ essential function, with an *rpoE*::Tn separation-of-function mutation (31) restored σ^S^-regulated promoter activity (Fig. 3B and C) and MBR (Fig. 1E) to Δ*gcvB* cells without increasing σ^S^ protein levels (see Fig. S2 in the supplemental material). The data imply that a too-active σ^E^ response inhibits the σ^S^ response and MBR, possibly via sigma factor competition for RNA polymerase (RNAP) (model below). Supporting this possibility, σ^E^ protein levels and activity were abnormally high in Δ*gcvB* cells (Fig. 5). We suggest that GcvB may promote membrane integrity and thus avert membrane stress response hyperinduction.

Our data demonstrate that GcvB promotes stress-inducible MBR and suggest a possible function for GcvB in E. coli in membrane maintenance. These data reinforce the importance and delicacy of stress response regulation of mutagenesis (39; for recent reviews, see references 2, 6, and 10).

### Model in which sigma factor competition reduces MBR in Δ*gcvB* mutant cells.

**FIG 7:**
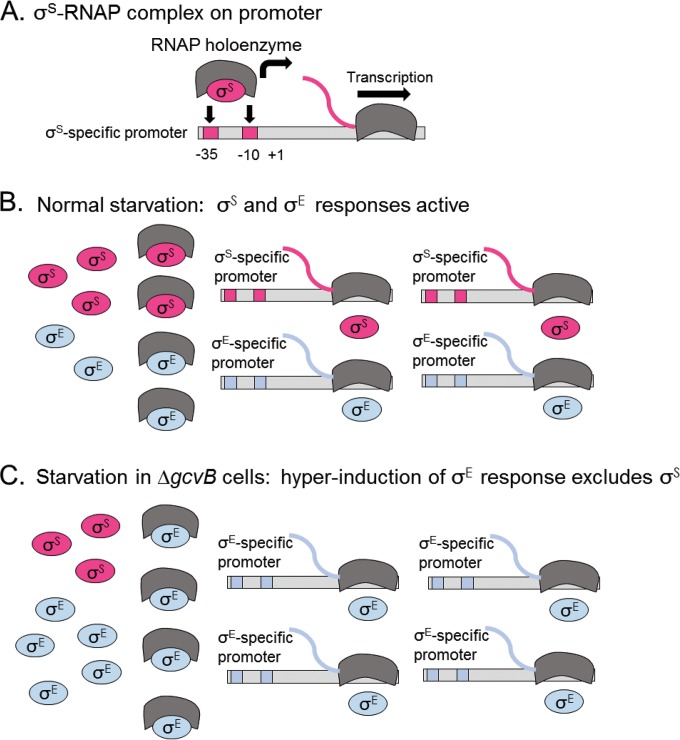
Model in which σ^E^ competition for RNA polymerase reduces the σ^S^ response and mutagenic break repair in Δ*gcvB* mutant cells. The observed σ^E^-dependent reductions of both MBR and σ^S^-dependent gene expression in Δ*gcvB* cells could result from competition of σ^E^ with σ^S^ for RNA polymerase (RNAP) (gray arc shapes) in cells that lack GcvB. (B) We suggest that sRNA GcvB is required for normal membrane integrity and thus keeps the σ^E^ response low in σ^S^-response-induced stationary cells. (C) In the absence of GcvB, excessive membrane stress is proposed to hyperactivate the σ^E^ response, producing increased σ^E^-RNAP complexes, at the expense of σ^S^-RNAP complexes. Reduced σ^S^-RNAP complexes could cause a reduced general stress response, which causes MBR deficiency (18, 19, 27, 28). The pink and blue lines represent σ^S^- and σ^E^-dependent transcripts, respectively.

In Fig. 7 we outline a possible model in which sigma factor competition for RNAP could promote the delicate regulatory balance between the σ^S^ and σ^E^ responses and thus modulate MBR in response to the presence or absence of GcvB sRNA. Sigma factor competition for RNAP has been implicated in shifting transcriptional patterns under various circumstances, and it is affected by both the number of sigma factors present in the cell and their affinity for RNAP (99, 100). σ^E^ has higher affinity for RNAP than σ^S^ has (101). Several bacterial sRNAs promote membrane integrity (67) (see the introduction), making it a reasonable hypothesis that GcvB suppresses the σ^E^ response (Fig. 5) because it, too, is needed for integrity of the cell membrane. GcvB would thus indirectly suppress hyper-σ^E^ response induction. We suggest that normally GcvB promotes membrane integrity and that when σ^S^ is induced during starvation, σ^S^-RNAP complexes can form, allowing activation of the σ^S^ response (Fig. 7B). We found previously that there is some σ^E^ response induction in E. coli under MBR starvation conditions (31), so we infer that normally during starvation both σ^S^ and σ^E^ responses are activated (Fig. 7B). We suggest that in Δ*gcvB* cells, membrane stress-promoted hyperinduction of σ^E^ blocks σ^S^ access to RNAP via competition (Fig. 7C). This model is supported by our findings that loss of σ^E^ response induction capability restores MBR and σ^S^-regulated promoter activity to Δ*gcvB* cells (Fig. 1E and 3B and C) without increasing σ^S^ protein levels (see Fig. S2 in the supplemental material). This model might also explain some of the acid sensitivity reported for Δ*gcvB* cells (65). Perhaps a hyperinduced σ^E^ response contributes to σ^S^ response depression and acid sensitivity of Δ*gcvB* cells in addition to the reduced production of σ^S^ (a σ^S^-LacZ fusion protein) observed in that study (65). We found that GcvB and a functional σ^E^ response both promote acid resistance (Fig. 6). The data suggest that both too much and too little σ^E^ response activity may result in sensitivity, and the data are not incompatible with the possibility that some of the acid sensitivity of E. coli Δ*gcvB* cells (65) results from a hyper-σ^E^ response. Other models are possible.

Which GcvB target gene(s) may affect membrane integrity under MBR starvation conditions is not known. A list of known and predicted targets of the GcvB sRNA is given in Table S3 in the supplemental material. An outer membrane protein gene, *ompF*, is an experimentally implicated GcvB target (102), and it might contribute to destabilization of the membrane when upregulated due to loss of GcvB. GcvB regulation of other genes on, and possibly not on, the lists in Table S3 might contribute to membrane integrity additionally or alternatively.

sRNAs play many and various roles in bacterial biology and across the tree of life, yet the functions of many sRNAs remain obscure, even in E. coli (68, 103, 104). GcvB is now implicated in membrane integrity and demonstrated to regulate mutagenesis. Other possible roles and the specific mechanism(s) of action of GcvB await future exploration.

## Supplementary Material

Supplemental material

## References

[1] Rosenberg SM, Shee C, Frisch RL, Hastings PJ 2012 Stress-induced mutation via DNA breaks in Escherichia coli: a molecular mechanism with implications for evolution and medicine. Bioessays 34:885–892. doi:10.1002/bies.201200050. 22911060 PMC3533179

[2] Rogers E, Correa R, Barreto B, Bravo Núñez MA, Minnick PJ, Vera Cruz D, Xia J, Hastings PJ, Rosenberg SM 2016 Double-strand-break repair, mutagenesis, and stress, p 185–195. *In* de Bruijn FJ (ed), Stress and environmental control of gene expression and adaptation in bacteria. Wiley & Sons Publisher, Hoboken, NJ.

[3] Foster PL 2007 Stress-induced mutagenesis in bacteria. Crit Rev Biochem Mol Biol 42:373–397. doi:10.1080/10409230701648494. 17917873 PMC2747772

[4] Robleto EA, Yasbin R, Ross C, Pedraza-Reyes M 2007 Stationary phase mutagenesis in B. subtilis: a paradigm to study genetic diversity programs in cells under stress. Crit Rev Biochem Mol Biol 42:327–339. doi:10.1080/10409230701597717. 17917870

[5] Saint-Ruf C, Pesut J, Sopta M, Matic I 2007 Causes and consequences of DNA repair activity modulation during stationary phase in Escherichia coli. Crit Rev Biochem Mol Biol 42:259–270. doi:10.1080/10409230701495599. 17687668

[6] Rogers E, Bravo Núñez MA, Hastings PJ, Rosenberg SM 2016 How a large gene network couples mutagenic DNA break repair to stress in Escherichia coli, p 570–576. de Bruijn FJ (ed), Stress and environmental control of gene expression and adaptation in bacteria. Wiley & Sons Publisher, Hoboken, NJ.

[7] Galhardo RS, Hastings PJ, Rosenberg SM 2007 Mutation as a stress response and the regulation of evolvability. Crit Rev Biochem Mol Biol 42:399–435. doi:10.1080/10409230701648502. 17917874 PMC3319127

[8] Shor E, Fox CA, Broach JR 2013 The yeast environmental stress response regulates mutagenesis induced by proteotoxic stress. PLoS Genet 9:e1003680. doi:10.1371/journal.pgen.1003680. 23935537 PMC3731204

[9] Bindra RS, Crosby ME, Glazer PM 2007 Regulation of DNA repair in hypoxic cancer cells. Cancer Metastasis Rev 26:249–260. doi:10.1007/s10555-007-9061-3. 17415527

[10] Fitzgerald DM, Hastings PJ, Rosenberg SM 16 9 2016 Stress-induced mutagenesis: implications in cancer and drug resistance. Annu Rev Cancer Biol 1:6.1–6.22. doi:10.1146/annurev-cancerbio-050216-121919. PMC5794033 29399660

[11] Ram Y, Hadany L 2012 The evolution of stress-induced hypermutation in asexual populations. Evolution 66:2315–2328. doi:10.1111/j.1558-5646.2012.01576.x. 22759304

[12] Ram Y, Hadany L 2014 Stress-induced mutagenesis and complex adaptation. Proc Biol Sci 281:20141025. doi:10.1098/rspb.2014.1025. 25143032 PMC4150318

[13] Bjedov I, Tenaillon O, Gerard B, Souza V, Denamur E, Radman M, Taddei F, Matic I 2003 Stress-induced mutagenesis in bacteria. Science 300:1404–1409. doi:10.1126/science.1082240. 12775833

[14] Cirz RT, Chin JK, Andes DR, de Crecy-Lagard V, Craig WA, Romesberg FE 2005 Inhibition of mutation and combating the evolution of antibiotic resistance. PLoS Biol 3:e176. doi:10.1371/journal.pbio.0030176. 15869329 PMC1088971

[15] Cirz RT, Romesberg FE 2007 Controlling mutation: intervening in evolution as a therapeutic strategy. Crit Rev Biochem Mol Biol 42:341–354. doi:10.1080/10409230701597741. 17917871

[16] Gutierrez A, Laureti L, Crussard S, Abida H, Rodriguez-Rojas A, Blazquez J, Baharoglu Z, Mazel D, Darfeuille F, Vogel J, Matic I 2013 Beta-lactam antibiotics promote bacterial mutagenesis via an RpoS-mediated reduction in replication fidelity. Nat Commun 4:1610. doi:10.1038/ncomms2607. 23511474 PMC3615471

[17] Forche A, Abbey D, Pisithkul T, Weinzierl MA, Ringstrom T, Bruck D, Petersen K, Berman J 2011 Stress alters rates and types of loss of heterozygosity in Candida albicans. mBio 2:e00129–11. doi:10.1128/mBio.00129-11. 21791579 PMC3143845

[18] Ponder RG, Fonville NC, Rosenberg SM 2005 A switch from high-fidelity to error-prone DNA double-strand break repair underlies stress-induced mutation. Mol Cell 19:791–804. doi:10.1016/j.molcel.2005.07.025. 16168374

[19] Shee C, Gibson JL, Darrow MC, Gonzalez C, Rosenberg SM 2011 Impact of a stress-inducible switch to mutagenic repair of DNA breaks on mutation in Escherichia coli. Proc Natl Acad Sci U S A 108:13659–13664. doi:10.1073/pnas.1104681108. 21808005 PMC3158223

[20] Shee C, Gibson JL, Rosenberg SM 2012 Two mechanisms produce mutation hotspots at DNA breaks in Escherichia coli. Cell Rep 2:714–721. doi:10.1016/j.celrep.2012.08.033. 23041320 PMC3607216

[21] Harris RS, Longerich S, Rosenberg SM 1994 Recombination in adaptive mutation. Science 264:258–260. doi:10.1126/science.8146657. 8146657

[22] Foster PL, Trimarchi JM, Maurer RA 1996 Two enzymes, both of which process recombination intermediates, have opposite effects on adaptive mutation in Escherichia coli. Genetics 142:25–37. 8770582 10.1093/genetics/142.1.25 PMC1206954

[23] Harris RS, Ross KJ, Rosenberg SM 1996 Opposing roles of the Holliday junction processing systems of Escherichia coli in recombination-dependent adaptive mutation. Genetics 142:681–691. 8849879 10.1093/genetics/142.3.681 PMC1207010

[24] McKenzie GJ, Lee PL, Lombardo MJ, Hastings PJ, Rosenberg SM 2001 SOS mutator DNA polymerase IV functions in adaptive mutation and not adaptive amplification. Mol Cell 7:571–579. doi:10.1016/S1097-2765(01)00204-0. 11463382

[25] Petrosino JF, Galhardo RS, Morales LD, Rosenberg SM 2009 Stress-induced beta-lactam antibiotic resistance mutation and sequences of stationary-phase mutations in the Escherichia coli chromosome. J Bacteriol 191:5881–5889. doi:10.1128/JB.00732-09. 19648247 PMC2747895

[26] Frisch RL, Su Y, Thornton PC, Gibson JL, Rosenberg SM, Hastings PJ 2010 Separate DNA Pol II- and Pol IV-dependent pathways of stress-induced mutation during double-strand-break repair in Escherichia coli are controlled by RpoS. J Bacteriol 192:4694–4700. doi:10.1128/JB.00570-10. 20639336 PMC2937414

[27] Layton JC, Foster PL 2003 Error-prone DNA polymerase IV is controlled by the stress-response sigma factor, RpoS, in Escherichia coli. Mol Microbiol 50:549–561. doi:10.1046/j.1365-2958.2003.03704.x. 14617178 PMC1237112

[28] Lombardo M-J, Aponyi I, Rosenberg SM 2004 General stress response regulator RpoS in adaptive mutation and amplification in Escherichia coli. Genetics 166:669–680. doi:10.1534/genetics.166.2.669. 15020458 PMC1470735

[29] Cairns J, Foster PL 1991 Adaptive reversion of a frameshift mutation in Escherichia coli. Genetics 128:695–701. 1916241 10.1093/genetics/128.4.695 PMC1204544

[30] McKenzie GJ, Harris RS, Lee PL, Rosenberg SM 2000 The SOS response regulates adaptive mutation. Proc Natl Acad Sci U S A 97:6646–6651. doi:10.1073/pnas.120161797. 10829077 PMC18688

[31] Gibson JL, Lombardo MJ, Thornton PC, Hu KH, Galhardo RS, Beadle B, Habib A, Magner DB, Frost LS, Herman C, Hastings PJ, Rosenberg SM 2010 The sigma(E) stress response is required for stress-induced mutation and amplification in Escherichia coli. Mol Microbiol 77:415–430. doi:10.1111/j.1365-2958.2010.07213.x. 20497332 PMC2909356

[32] Wimberly H, Shee C, Thornton PC, Sivaramakrishnan P, Rosenberg SM, Hastings PJ 2013 R-loops and nicks initiate DNA breakage and genome instability in non-growing Escherichia coli. Nat Commun 4:2115. doi:10.1038/ncomms3115. 23828459 PMC3715873

[33] Galhardo RS, Do R, Yamada M, Friedberg EC, Hastings PJ, Nohmi T, Rosenberg SM 2009 DinB upregulation is the sole role of the SOS response in stress-induced mutagenesis in Escherichia coli. Genetics 182:55–68. doi:10.1534/genetics.109.100735. 19270270 PMC2674841

[34] Battesti A, Majdalani N, Gottesman S 2011 The RpoS-mediated general stress response in Escherichia coli. Annu Rev Microbiol 65:189–213. doi:10.1146/annurev-micro-090110-102946. 21639793 PMC7356644

[35] Hengge-Aronis R 2002 Signal transduction and regulatory mechanisms involved in control of the sigma(S) (RpoS) subunit of RNA polymerase. Microbiol Mol Biol Rev 66:373–395. doi:10.1128/MMBR.66.3.373-395.2002. 12208995 PMC120795

[36] Motamedi MR, Szigety SK, Rosenberg SM 1999 Double-strand-break repair recombination in Escherichia coli: physical evidence for a DNA replication mechanism in vivo. Genes Dev 13:2889–2903. doi:10.1101/gad.13.21.2889. 10557215 PMC317119

[37] Pomerantz RT, Kurth I, Goodman MF, O'Donnell ME 2013 Preferential D-loop extension by a translesion DNA polymerase underlies error-prone recombination. Nat Struct Mol Biol 20:748–755. doi:10.1038/nsmb.2573. 23686288 PMC3685420

[38] Maharjan R, Ferenci T 2015 Mutational signatures indicative of environmental stress in bacteria. Mol Biol Evol 32:380–391. doi:10.1093/molbev/msu306. 25389207

[39] Al Mamun AA, Lombardo MJ, Shee C, Lisewski AM, Gonzalez C, Lin D, Nehring RB, Saint-Ruf C, Gibson JL, Frisch RL, Lichtarge O, Hastings PJ, Rosenberg SM 2012 Identity and function of a large gene network underlying mutagenic repair of DNA breaks. Science 338:1344–1348. doi:10.1126/science.1226683. 23224554 PMC3782309

[40] Kajitani M, Ishihama A 1991 Identification and sequence determination of the host factor gene for bacteriophage Q beta. Nucleic Acids Res 19:1063–1066. doi:10.1093/nar/19.5.1063. 2020545 PMC333781

[41] Brennan RG, Link TM 2007 Hfq structure, function and ligand binding. Curr Opin Microbiol 10:125–133. doi:10.1016/j.mib.2007.03.015. 17395525

[42] Chao Y, Vogel J 2010 The role of Hfq in bacterial pathogens. Curr Opin Microbiol 13:24–33. doi:10.1016/j.mib.2010.01.001. 20080057

[43] Sittka A, Pfeiffer V, Tedin K, Vogel J 2007 The RNA chaperone Hfq is essential for the virulence of Salmonella typhimurium. Mol Microbiol 63:193–217. doi:10.1111/j.1365-2958.2006.05489.x. 17163975 PMC1810395

[44] Simonsen KT, Nielsen G, Bjerrum JV, Kruse T, Kallipolitis BH, Moller-Jensen J 2011 A role for the RNA chaperone Hfq in controlling adherent-invasive Escherichia coli colonization and virulence. PLoS One 6:e16387. doi:10.1371/journal.pone.0016387. 21298102 PMC3027648

[45] Christiansen JK, Larsen MH, Ingmer H, Sogaard-Andersen L, Kallipolitis BH 2004 The RNA-binding protein Hfq of Listeria monocytogenes: role in stress tolerance and virulence. J Bacteriol 186:3355–3362. doi:10.1128/JB.186.11.3355-3362.2004. 15150220 PMC415768

[46] Geng J, Song Y, Yang L, Feng Y, Qiu Y, Li G, Guo J, Bi Y, Qu Y, Wang W, Wang X, Guo Z, Yang R, Han Y 2009 Involvement of the post-transcriptional regulator Hfq in Yersinia pestis virulence. PLoS One 4:e6213. doi:10.1371/journal.pone.0006213. 19593436 PMC2704395

[47] Ding Y, Davis BM, Waldor MK 2004 Hfq is essential for Vibrio cholerae virulence and downregulates sigma expression. Mol Microbiol 53:345–354. doi:10.1111/j.1365-2958.2004.04142.x. 15225327

[48] Sonnleitner E, Hagens S, Rosenau F, Wilhelm S, Habel A, Jager KE, Blasi U 2003 Reduced virulence of a hfq mutant of Pseudomonas aeruginosa O1. Microb Pathog 35:217–228. doi:10.1016/S0882-4010(03)00149-9. 14521880

[49] Wang MC, Chien HF, Tsai YL, Liu MC, Liaw SJ 2014 The RNA chaperone Hfq is involved in stress tolerance and virulence in uropathogenic Proteus mirabilis. PLoS One 9:e85626. doi:10.1371/journal.pone.0085626. 24454905 PMC3893223

[50] Valentin-Hansen P, Eriksen M, Udesen C 2004 The bacterial Sm-like protein Hfq: a key player in RNA transactions. Mol Microbiol 51:1525–1533. doi:10.1111/j.1365-2958.2003.03935.x. 15009882

[51] Vogel J, Luisi BF 2011 Hfq and its constellation of RNA. Nat Rev Microbiol 9:578–589. doi:10.1038/nrmicro2615. 21760622 PMC4615618

[52] De Lay N, Schu DJ, Gottesman S 2013 Bacterial small RNA-based negative regulation: Hfq and its accomplices. J Biol Chem 288:7996–8003. doi:10.1074/jbc.R112.441386. 23362267 PMC3605619

[53] Frohlich KS, Vogel J 2009 Activation of gene expression by small RNA. Curr Opin Microbiol 12:674–682. doi:10.1016/j.mib.2009.09.009. 19880344

[54] McCullen CA, Benhammou JN, Majdalani N, Gottesman S 2010 Mechanism of positive regulation by DsrA and RprA small noncoding RNAs: pairing increases translation and protects rpoS mRNA from degradation. J Bacteriol 192:5559–5571. doi:10.1128/JB.00464-10. 20802038 PMC2953674

[55] Chen S, Lesnik EA, Hall TA, Sampath R, Griffey RH, Ecker DJ, Blyn LB 2002 A bioinformatics based approach to discover small RNA genes in the Escherichia coli genome. Biosystems 65:157–177. doi:10.1016/S0303-2647(02)00013-8. 12069726

[56] Wassarman KM, Repoila F, Rosenow C, Storz G, Gottesman S 2001 Identification of novel small RNAs using comparative genomics and microarrays. Genes Dev 15:1637–1651. doi:10.1101/gad.901001. 11445539 PMC312727

[57] Vogel J, Bartels V, Tang TH, Churakov G, Slagter-Jager JG, Huttenhofer A, Wagner EG 2003 RNomics in Escherichia coli detects new sRNA species and indicates parallel transcriptional output in bacteria. Nucleic Acids Res 31:6435–6443. doi:10.1093/nar/gkg867. 14602901 PMC275561

[58] Argaman L, Hershberg R, Vogel J, Bejerano G, Wagner EG, Margalit H, Altuvia S 2001 Novel small RNA-encoding genes in the intergenic regions of Escherichia coli. Curr Biol 11:941–950. doi:10.1016/S0960-9822(01)00270-6. 11448770

[59] Zhang A, Wassarman KM, Rosenow C, Tjaden BC, Storz G, Gottesman S 2003 Global analysis of small RNA and mRNA targets of Hfq. Mol Microbiol 50:1111–1124. doi:10.1046/j.1365-2958.2003.03734.x. 14622403

[60] Urbanowski ML, Stauffer LT, Stauffer GV 2000 The gcvB gene encodes a small untranslated RNA involved in expression of the dipeptide and oligopeptide transport systems in Escherichia coli. Mol Microbiol 37:856–868. doi:10.1046/j.1365-2958.2000.02051.x. 10972807

[61] Pulvermacher SC, Stauffer LT, Stauffer GV 2009 Role of the Escherichia coli Hfq protein in GcvB regulation of oppA and dppA mRNAs. Microbiology 155:115–123. doi:10.1099/mic.0.023432-0. 19118352

[62] Pulvermacher SC, Stauffer LT, Stauffer GV 2009 The small RNA GcvB regulates sstT mRNA expression in Escherichia coli. J Bacteriol 191:238–248. doi:10.1128/JB.00915-08. 18952787 PMC2612445

[63] Pulvermacher SC, Stauffer LT, Stauffer GV 2009 Role of the sRNA GcvB in regulation of cycA in Escherichia coli. Microbiology 155:106–114. doi:10.1099/mic.0.023598-0. 19118351

[64] Sharma CM, Papenfort K, Pernitzsch SR, Mollenkopf HJ, Hinton JC, Vogel J 2011 Pervasive post-transcriptional control of genes involved in amino acid metabolism by the Hfq-dependent GcvB small RNA. Mol Microbiol 81:1144–1165. doi:10.1111/j.1365-2958.2011.07751.x. 21696468

[65] Jin Y, Watt RM, Danchin A, Huang JD 2009 Small noncoding RNA GcvB is a novel regulator of acid resistance in Escherichia coli. BMC Genomics 10:165. doi:10.1186/1471-2164-10-165. 19379489 PMC2676305

[66] Vogel J, Papenfort K 2006 Small non-coding RNAs and the bacterial outer membrane. Curr Opin Microbiol 9:605–611. doi:10.1016/j.mib.2006.10.006. 17055775

[67] Guillier M, Gottesman S, Storz G 2006 Modulating the outer membrane with small RNAs. Genes Dev 20:2338–2348. doi:10.1101/gad.1457506. 16951250

[68] Gottesman S, Storz G 2011 Bacterial small RNA regulators: versatile roles and rapidly evolving variations. Cold Spring Harb Perspect Biol 3:a003798. doi:10.1101/cshperspect.a003798. 20980440 PMC3225950

[69] Tjaden B, Saxena RM, Stolyar S, Haynor DR, Kolker E, Rosenow C 2002 Transcriptome analysis of Escherichia coli using high-density oligonucleotide probe arrays. Nucleic Acids Res 30:3732–3738. doi:10.1093/nar/gkf505. 12202758 PMC137427

[70] Papenfort K, Pfeiffer V, Mika F, Lucchini S, Hinton JC, Vogel J 2006 SigmaE-dependent small RNAs of Salmonella respond to membrane stress by accelerating global omp mRNA decay. Mol Microbiol 62:1674–1688. doi:10.1111/j.1365-2958.2006.05524.x. 17427289 PMC1804206

[71] Thompson KM, Rhodius VA, Gottesman S 2007 SigmaE regulates and is regulated by a small RNA in Escherichia coli. J Bacteriol 189:4243–4256. doi:10.1128/JB.00020-07. 17416652 PMC1913397

[72] Johansen J, Rasmussen AA, Overgaard M, Valentin-Hansen P 2006 Conserved small non-coding RNAs that belong to the sigmaE regulon: role in down-regulation of outer membrane proteins. J Mol Biol 364:1–8. doi:10.1016/j.jmb.2006.09.004. 17007876

[73] Rasmussen AA, Eriksen M, Gilany K, Udesen C, Franch T, Petersen C, Valentin-Hansen P 2005 Regulation of ompA mRNA stability: the role of a small regulatory RNA in growth phase-dependent control. Mol Microbiol 58:1421–1429. doi:10.1111/j.1365-2958.2005.04911.x. 16313626

[74] Udekwu KI, Darfeuille F, Vogel J, Reimegard J, Holmqvist E, Wagner EG 2005 Hfq-dependent regulation of OmpA synthesis is mediated by an antisense RNA. Genes Dev 19:2355–2366. doi:10.1101/gad.354405. 16204185 PMC1240044

[75] Coornaert A, Lu A, Mandin P, Springer M, Gottesman S, Guillier M 2010 MicA sRNA links the PhoP regulon to cell envelope stress. Mol Microbiol 76:467–479. doi:10.1111/j.1365-2958.2010.07115.x. 20345657 PMC2925231

[76] Gogol EB, Rhodius VA, Papenfort K, Vogel J, Gross CA 2011 Small RNAs endow a transcriptional activator with essential repressor functions for single-tier control of a global stress regulon. Proc Natl Acad Sci U S A 108:12875–12880. doi:10.1073/pnas.1109379108. 21768388 PMC3150882

[77] Guo MS, Updegrove TB, Gogol EB, Shabalina SA, Gross CA, Storz G 2014 MicL, a new sigmaE-dependent sRNA, combats envelope stress by repressing synthesis of Lpp, the major outer membrane lipoprotein. Genes Dev 28:1620–1634. doi:10.1101/gad.243485.114. 25030700 PMC4102768

[78] Guisbert E, Rhodius VA, Ahuja N, Witkin E, Gross CA 2007 Hfq modulates the sigmaE-mediated envelope stress response and the sigma32-mediated cytoplasmic stress response in Escherichia coli. J Bacteriol 189:1963–1973. doi:10.1128/JB.01243-06. 17158661 PMC1855744

[79] Datsenko KA, Wanner BL 2000 One-step inactivation of chromosomal genes in Escherichia coli K-12 using PCR products. Proc Natl Acad Sci U S A 97:6640–6645. doi:10.1073/pnas.120163297. 10829079 PMC18686

[80] Miller JH 1992 A short course in bacterial genetics: a laboratory manual and handbook for Escherichia coli and related bacteria. Cold Spring Harbor Laboratory Press, Plainview, NY.

[81] Torkelson J, Harris RS, Lombardo MJ, Nagendran J, Thulin C, Rosenberg SM 1997 Genome-wide hypermutation in a subpopulation of stationary-phase cells underlies recombination-dependent adaptive mutation. EMBO J 16:3303–3311. doi:10.1093/emboj/16.11.3303. 9214645 PMC1169946

[82] Pennington JM, Rosenberg SM 2007 Spontaneous DNA breakage in single living Escherichia coli cells. Nat Genet 39:797–802. doi:10.1038/ng2051. 17529976 PMC2856310

[83] Nehring RB, Gu F, Lin HY, Gibson JL, Blythe MJ, Wilson R, Bravo Nunez MA, Hastings PJ, Louis EJ, Frisch RL, Hu JC, Rosenberg SM 2016 An ultra-dense library resource for rapid deconvolution of mutations that cause phenotypes in Escherichia coli. Nucleic Acids Res 44:e41. doi:10.1093/nar/gkv1131. 26578563 PMC4797258

[84] Nguyen LH, Jensen DB, Thompson NE, Gentry DR, Burgess RR 1993 In vitro functional characterization of overproduced Escherichia coli katF/rpoS gene product. Biochemistry 32:11112–11117. doi:10.1021/bi00092a021. 8218173

[85] Ades SE, Connolly LE, Alba BM, Gross CA 1999 The Escherichia coli sigma(E)-dependent extracytoplasmic stress response is controlled by the regulated proteolysis of an anti-sigma factor. Genes Dev 13:2449–2461. doi:10.1101/gad.13.18.2449. 10500101 PMC317020

[86] McKenzie GJ, Lombardo MJ, Rosenberg SM 1998 Recombination-dependent mutation in Escherichia coli occurs in stationary phase. Genetics 149:1163–1165. 9735004 10.1093/genetics/149.2.1163 PMC1460184

[87] Rosenberg SM 2001 Evolving responsively: adaptive mutation. Nat Rev Genet 2:504–515. doi:10.1038/35080556. 11433357

[88] Rouviere PE, De Las Penas A, Mecsas J, Lu CZ, Rudd KE, Gross CA 1995 *rpoE*, the gene encoding the second heat-shock sigma factor, sigma E, in Escherichia coli. EMBO J 14:1032–1042. 7889934 10.1002/j.1460-2075.1995.tb07084.x PMC398175

[89] De Las Penas A, Connolly L, Gross CA 1997 SigmaE is an essential sigma factor in Escherichia coli. J Bacteriol 179:6862–6864. 9352942 10.1128/jb.179.21.6862-6864.1997 PMC179621

[90] Alba BM, Gross CA 2004 Regulation of the Escherichia coli sigma-dependent envelope stress response. Mol Microbiol 52:613–619. doi:10.1111/j.1365-2958.2003.03982.x. 15101969

[91] Loewen PC 1984 Isolation of catalase-deficient Escherichia coli mutants and genetic mapping of katE, a locus that affects catalase activity. J Bacteriol 157:622–626. 6319370 10.1128/jb.157.2.622-626.1984 PMC215291

[92] Mecsas J, Rouviere PE, Erickson JW, Donohue TJ, Gross CA 1993 The activity of sigma E, an Escherichia coli heat-inducible sigma-factor, is modulated by expression of outer membrane proteins. Genes Dev 7:2618–2628. doi:10.1101/gad.7.12b.2618. 8276244

[93] Akerlund T, Nordstrom K, Bernander R 1995 Analysis of cell size and DNA content in exponentially growing and stationary-phase batch cultures of Escherichia coli. J Bacteriol 177:6791–6797. 7592469 10.1128/jb.177.23.6791-6797.1995 PMC177544

[94] Radman M 1975 SOS repair hypothesis: phenomenology of an inducible DNA repair which is accompanied by mutagenesis, p 355–367. *In* Hanawalt P, Setlow RB (ed), Molecular mechanisms for repair of DNA. Plenum Press, New York, NY. 10.1007/978-1-4684-2895-7_48 1103845

[95] Witkin EM 1976 Ultraviolet mutagenesis and inducible DNA repair in Escherichia coli. Bacteriol Rev 40:869–907. 795416 10.1128/br.40.4.869-907.1976 PMC413988

[96] Vijayakumar SR, Kirchhof MG, Patten CL, Schellhorn HE 2004 RpoS-regulated genes of Escherichia coli identified by random *lacZ* fusion mutagenesis. J Bacteriol 186:8499–8507. doi:10.1128/JB.186.24.8499-8507.2004. 15576800 PMC532425

[97] Hryckowian AJ, Battesti A, Lemke JJ, Meyer ZC, Welch RA 2014 IraL is an RssB anti-adaptor that stabilizes RpoS during logarithmic phase growth in Escherichia coli and Shigella. mBio 5:e01043–14. doi:10.1128/mBio.01043-14. 24865554 PMC4045071

[98] Gonzalez C 2009 A hypermutable cell subpopulation in stress-induced mutagenesis. PhD thesis Baylor College of Medicine, Houston, TX.

[99] Malik S, Zalenskaya K, Goldfarb A 1987 Competition between sigma factors for core RNA polymerase. Nucleic Acids Res 15:8521–8530. doi:10.1093/nar/15.20.8521. 3313282 PMC306375

[100] Kolesky S, Ouhammouch M, Brody EN, Geiduschek EP 1999 Sigma competition: the contest between bacteriophage T4 middle and late transcription. J Mol Biol 291:267–281. doi:10.1006/jmbi.1999.2953. 10438620

[101] Maeda H, Fujita N, Ishihama A 2000 Competition among seven Escherichia coli sigma subunits: relative binding affinities to the core RNA polymerase. Nucleic Acids Res 28:3497–3503. doi:10.1093/nar/28.18.3497. 10982868 PMC110723

[102] Melamed S, Peer A, Faigenbaum-Romm R, Gatt YE, Reiss N, Bar A, Altuvia Y, Argaman L, Margalit H 2016 Global mapping of small RNA-target interactions in bacteria. Mol Cell 63:884–897. doi:10.1016/j.molcel.2016.07.026. 27588604 PMC5145812

[103] Waters LS, Storz G 2009 Regulatory RNAs in bacteria. Cell 136:615–628. doi:10.1016/j.cell.2009.01.043. 19239884 PMC3132550

[104] Livny J, Waldor MK 2007 Identification of small RNAs in diverse bacterial species. Curr Opin Microbiol 10:96–101. doi:10.1016/j.mib.2007.03.005. 17383222

[105] Ivanova AB, Glinsky GV, Eisenstark A 1997 Role of rpoS regulon in resistance to oxidative stress and near-UV radiation in delta oxyR suppressor mutants of Escherichia coli. Free Radic Biol Med 23:627–636. doi:10.1016/S0891-5849(97)00013-0. 9215808

[106] Ades SE, Grigorova IL, Gross CA 2003 Regulation of the alternative sigma factor E during initiation, adaptation, and shutoff of the extracytoplasmic heat shock response in Escherichia coli. J Bacteriol 185:2512–2519. doi:10.1128/JB.185.8.2512-2519.2003. 12670975 PMC152616

[107] Dri AM, Rouviere-Yaniv J, Moreau PL 1991 Inhibition of cell division in hupA hupB mutant bacteria lacking HU protein. J Bacteriol 173:2852–2863. 2019558 10.1128/jb.173.9.2852-2863.1991 PMC207866

[108] Dutreix M, Moreau PL, Bailone A, Galibert F, Battista JR, Walker GC, Devoret R 1989 New recA mutations that dissociate the various RecA protein activities in Escherichia coli provide evidence for an additional role for RecA protein in UV mutagenesis. J Bacteriol 171:2415–2423. 2651400 10.1128/jb.171.5.2415-2423.1989 PMC209916

[109] Baba T, Ara T, Hasegawa M, Takai Y, Okumura Y, Baba M, Datsenko KA, Tomita M, Wanner BL, Mori H 2006 Construction of Escherichia coli K-12 in-frame, single-gene knockout mutants: the Keio collection. Mol Syst Biol 2:2006–0008. doi:10.1038/msb4100050. PMC1681482 16738554

[110] Cherepanov PP, Wackernagel W 1995 Gene disruption in Escherichia coli: TcR and KmR cassettes with the option of Flp-catalyzed excision of the antibiotic-resistance determinant. Gene 158:9–14. doi:10.1016/0378-1119(95)00193-A. 7789817

