# Supplementary material for: The Small RNA GcvB Promotes Mutagenic Break Repair by Opposing the Membrane Stress Response
Source: J Bacteriol. 2016 Nov 18;198(24):3296–308. doi: 10.1128/JB.00555-16 (PMC5116933; doi:10.1128/JB.00555-16)
Supplement: Supplemental material [file supp_198_24_3296__index.html]

Supplemental material 

# The Small RNA GcvB Promotes Mutagenic Break Repair by Opposing the Membrane Stress Response

## Supplemental material

**Files in this Data Supplement:**

- Supplemental file 1 -

  Tables S1 (Genome coordinates of nonpolar sRNA gene deletions), S2 (*rssB* epistasis to Δ*gcvB* in MBR), and S3 (Targets of GcvB) and Fig. S1

  PDF, 621K
