# Supplementary material for: The Small RNA GcvB Promotes Mutagenic Break Repair by Opposing the Membrane Stress Response
Source: J Bacteriol. 2016 Nov 18;198(24):3296–308. doi: 10.1128/JB.00555-16 (PMC5116933; doi:10.1128/JB.00555-16)
Supplement: Supplemental material [file JB.00555-16_zjb999094258so1.pdf]

SUPPLEMENTAL INFORMATION

TABLE S1 Genome coordinates of nine non-polar sRNA-gene deletions created

New allele[a]	Basepairs deleted[b]
Δ*cyaR*	2,165,179 – 2,165,263
Δ*dsrA*	2,023,212 – 2,023,376
Δ*gcvB*	2,940,679 – 2,940,961
Δ*micF*	2,311,065 – 2,311,237
Δ*oxyS*	4,156,267 – 4,156,457
Δ*rprA*	1,768,358 – 1,768,539
Δ*rybB*	887,161 – 887,319
Δ*ryhB*	3,578,911 – 3,579,079
Δ*spf*	4,047,883 – 4,048,069

[a] Nonpolar deletions of the sRNA genes were recombineered (1) using PCR with pKD3 (1) as a template, to replace the *E. coli* sequences indicated with the chloramphenicol acetyl transferase gene flanked by FRT sites: FRT*cat*FRT. The *cat* gene and one FRT were removed by Flp recombinase for all final strains used in experiments. These are non-polar because they have no intervening transcription unit that might affect expression of downstream genes.

[b]Coordinates correspond to the MG1655 genome positions (NC_000913.2) of the sequences deleted.

TABLE S2 σ^S negative-regulatory gene *rssB* is epistatic to Δ*gcvB* in MBR

Relevant genotype	Mean mutation rate	*p* value (mutation rate relative to WT)	Mean fold-change in mutation rate over WT	Mean fold-change in mutation rate over Δ*gcvB*	<u>Δ*rssB* Δ*gcvB* / Δ*gcvB*</u> Δ*rssB*/WT
WT	33 ± 11		1	8.1 ± 1.4	
Δ*rpoS*	4.5 ± 0.4	0.05	0.18 ± 0.04	1.2 ± 0.2	
Δ*gcvB*	3.8 ± 0.8	0.05	0.13 ± 0.02	1	
Δ*rssB*	120 ± 19	0.006	4.4 ± 0.7	33 ± 3.7	
Δ*rssB* Δ*gcvB*	130 ± 31	0.04	**4.3 ± 1**	**31 ± 2.5**	**7.9 ± 1.7**

Epistasis analysis: quantification of single and double mutants, fold change in mutation rate. The mutation rate (Lac^+ cfu/10^8 cells/day, mean ± SEM) is decreased in Δ*gcvB* cells. The fold changes of each strain compared with either wild-type (SMR4562) or Δ*gcvB* (SMR20238) were calculated for each experiment and averaged. Mean fold-difference of ratios of mutation rate for double mutants relative to control were calculated by taking the ratio of Δ*rssB* Δ*gcvB* to Δ*gcvB* and dividing by the ratio of Δ*rssB* to WT for all three experiments averaged together. A value greater than one indicates suppression of the MBR defect by Δ*rssB* (upregulation of σ^S). The ratio of mutation rate for the double mutant was statistically significant and thus indicates that the MBR defect in Δ*gcvB* was relieved by Δ*rssB*. The data imply that GcvB promotes MBR by allowing the σ^S response and is not needed when σ^S is otherwise upregulated.

TABLE S3 Known and predicted targets of GcvB

A. GcvB targets supported by experimental evidence

Target	Experimental evidence	Computationally predicted [a]	References
argT	RNA:RNA interaction	yes	(2)
aroP	RNA:RNA interaction	no	(2)
bamD	RNA:RNA interaction	no	(2)
cfa	RNA:RNA interaction	no	(2)
csgD	regulation	no	(3, 4)
cstA	RNA:RNA interaction	no	(2)
cycA	regulation, RNA:RNA interaction	yes	(2, 5)
cysB	RNA:RNA interaction	no	(2)
dppA	regulation, RNA:RNA interaction	yes	(2, 6, 7)
ebgR	RNA:RNA interaction	no	(2)
fliZ	RNA:RNA interaction	no	(2)
gatY	RNA:RNA interaction	no	(2)
gdhA	RNA:RNA interaction	yes	(2)
glmS	RNA:RNA interaction	no	(2)
glpC	RNA:RNA interaction	no	(2)
gltI	RNA:RNA interaction	yes	(2)
gltP	RNA:RNA interaction	no	(2)
hdeAB	regulation	no	(8)
hisQ	RNA:RNA interaction	no	(2)
Icd	RNA:RNA interaction	no	(2)
ilvM	RNA:RNA interaction	no	(2)
insI-1	RNA:RNA interaction	no	(2)
kgtP	RNA:RNA interaction	no	(2)
lipA	RNA:RNA interaction	no	(2)
livJ	RNA:RNA interaction	yes	(2)
livK	RNA:RNA interaction	yes	(2)
lrp	regulation	yes	(9)
maeB	RNA:RNA interaction	no	(2)
map	RNA:RNA interaction	no	(2)
mcrB	RNA:RNA interaction	no	(2)
nplA	RNA:RNA interaction	no	(2)
ompF	RNA:RNA interaction	no	(2)
oppA	regulation, RNA:RNA interaction	yes	(2, 6, 7)
panD	RNA:RNA interaction	yes	(2)
phoP	regulation	no	(2)
prmB	RNA:RNA interaction	no	(2)
purU	RNA:RNA interaction	no	(2)
raiA	RNA:RNA interaction	no	(2)
rhlB	RNA:RNA interaction	no	(2)
rihA	RNA:RNA interaction	no	(2)
rppH	RNA:RNA interaction	no	(2)
sdhE	RNA:RNA interaction	no	(2)

serA	RNA:RNA interaction	yes	(2)
sstT	regulation, RNA:RNA interaction	no	(2, 10)
thrL	RNA:RNA interaction	no	(2)
waaP	RNA:RNA interaction	no	(2)
yabQ	RNA:RNA interaction	no	(2)
yagU	RNA:RNA interaction	no	(2)
ydeE	RNA:RNA interaction	no	(2)
yggX	RNA:RNA interaction	no	(2)
yhjE	RNA:RNA interaction	no	(2)
yidF	RNA:RNA interaction	no	(2)
yifK	RNA:RNA interaction	yes	(2)
ylcI	RNA:RNA interaction	no	(2)
ysgA	RNA:RNA interaction	yes	(2)
zapE	RNA:RNA interaction	no	(2)
zitB	RNA:RNA interaction	no	(2)

B. Top computationally predicted GcvB targets without experimental support [b]

	IntaRNA (11)		TargetRNA2 (12)	
**Target**	**ΔG (kJ/mol)**	**p-value**	**ΔG (kJ/mol)**	**p-value**
abgT	-19.72	0.0008	-20.81	0.0000
aroC	-18.03	0.0024	-15.97	0.0000
asnB	-22.74	0.0001	-15.84	0.0000
dcyD	-17.94	0.0025	-20.17	0.0000
mltC	-16.48	0.0060	-15.67	0.0000
panB	-16.30	0.0067	-16.08	0.0000
trpE	-18.13	0.0023	-18.60	0.0000
yafX	-19.05	0.0013	-16.10	0.0000
ilvC	-18.84	0.0014	-14.45	0.0010
ybiC	-16.39	0.0064	-14.59	0.0010
ilvE	-15.87	0.0085	-15.15	0.0010
yhjV	-14.53	0.0176	-14.80	0.0010
dtpB	-14.02	0.0230	-14.17	0.0010
ybdH	-16.49	0.0060	-12.90	0.0040
yadD	-15.48	0.0106	-13.26	0.0030
yahI	-18.55	0.0017	-10.36	0.0190
yeiG	-15.08	0.0132	-12.37	0.0060
yfbL	-14.43	0.0186	-12.72	0.0050
fliY	-14.08	0.0223	-12.50	0.0050
tynA	-14.20	0.0210	-12.46	0.0060
metF	-16.73	0.0052	-9.19	0.0340
sucC	-13.78	0.0262	-8.65	0.0430

[a] Indication of whether experimentally determined GcvB targets were computationally predicted using the same criteria described in part B.
[b] GcvB targets were computationally predicted using the IntaRNA and TargetRNA webservers and targets predicted by both algorithms were considered top candidates and

listed in part B, if not supported by experimental evidence.
[c] Predicted change in free energy with predicted RNA-RNA binding (a measure of affinity with lower numbers reflecting better affinity).

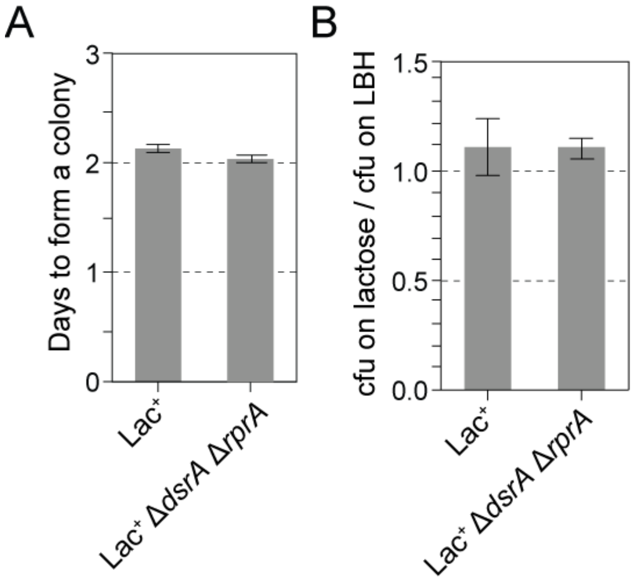

FIG S1 Lac⁺ Δ*dsrA* Δ*rprA* cells form colonies normally under precise reconstructions of experimental conditions, on selection plates with 10^9 non-revertible Δ*lac* neighbor cells. (A) Normal speed of colony formation of Δ*dsrA* Δ*rprA* cells, and (B) similar efficiencies of colony formation under selective conditions, compared with cfu on rich (LBH) medium without neighbor cells. Mean ± SEM of three experiments. Strains SMR3856 and SMR22960 plated with Δ*lac* FC29 neighbor cells in panel A and also in panel B on the lactose medium.

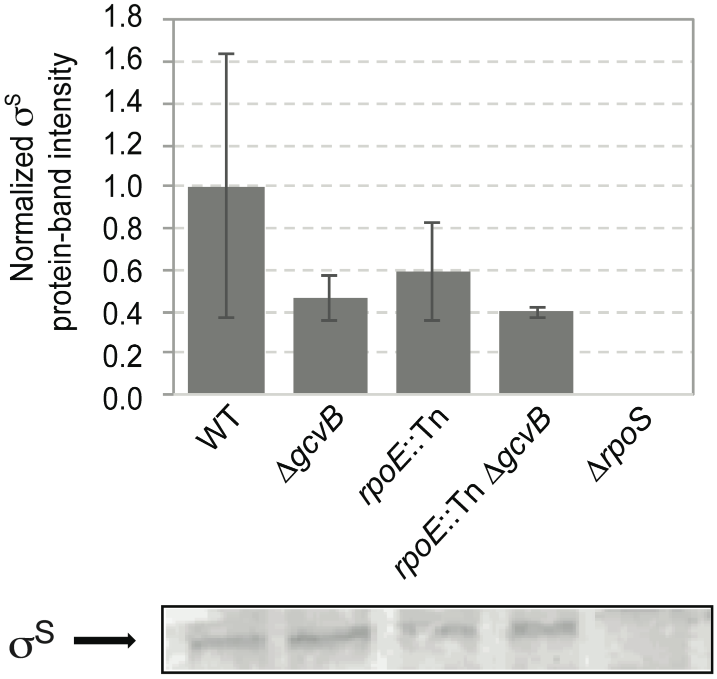

FIG S2 Blocking the σ^E response with the *rpoE2072*::Tn*10*dCam allele does not increase σ^S protein levels in starving Δ*gcvB* cells. Western analyses show that σ^S protein levels in stationary phase cells prepared and starved as for the Tet assay are not increased by the *rpoE2072*::Tn*10*dCam (*rpoE*::Tn) mutation. Top: mean ± range, 2 quantified immunoblots normalized to WT. Bottom: representative immunoblot. Strains left to right: SMR10866; SMR21633; SMR10854; SMR22074; SMR10862.

SUPPLEMENTAL REFERENCES

1. **Datsenko KA, Wanner BL.** 2000. One-step inactivation of chromosomal genes in *Escherichia coli* K-12 using PCR products. Proc Natl Acad Sci U S A **97:**6640-6645.
2. **Melamed S, Peer A, Faigenbaum-Romm R, Gatt YE, Reiss N, Bar A, Altuvia Y, Argaman L, Margalit H.** 2016. Global Mapping of Small RNA-Target Interactions in Bacteria. Mol Cell **63:**884-897.
3. **Jorgensen MG, Nielsen JS, Boysen A, Franch T, Moller-Jensen J, Valentin-Hansen P.** 2012. Small regulatory RNAs control the multi-cellular adhesive lifestyle of Escherichia coli. Mol Microbiol **84:**36-50.
4. **Mika F, Hengge R.** 2014. Small RNAs in the control of RpoS, CsgD, and biofilm architecture of Escherichia coli. RNA Biol **11:**494-507.
5. **Pulvermacher SC, Stauffer LT, Stauffer GV.** 2009. Role of the sRNA GcvB in regulation of cycA in *Escherichia coli*. Microbiology **155:**106-114.
6. **Pulvermacher SC, Stauffer LT, Stauffer GV.** 2008. The role of the small regulatory RNA GcvB in GcvB/mRNA posttranscriptional regulation of oppA and dppA in Escherichia coli. FEMS Microbiol Lett **281:**42-50.
7. **Pulvermacher SC, Stauffer LT, Stauffer GV.** 2009. Role of the Escherichia coli Hfq protein in GcvB regulation of oppA and dppA mRNAs. Microbiology **155:**115-123.
8. **Stauffer LT, Stauffer GV.** 2012. Antagonistic Roles for GcvA and GcvB in hdeAB Expression in Escherichia coli. ISRN Microbiol **2012:**697308.
9. **Modi SR, Camacho DM, Kohanski MA, Walker GC, Collins JJ.** 2011. Functional characterization of bacterial sRNAs using a network biology approach. Proc Natl Acad Sci U S A **108:**15522-15527.
10. **Pulvermacher SC, Stauffer LT, Stauffer GV.** 2009. The small RNA GcvB regulates sstT mRNA expression in *Escherichia coli*. J Bacteriol **191:**238-248.
11. **Busch A, Richter AS, Backofen R.** 2008. IntaRNA: efficient prediction of bacterial sRNA targets incorporating target site accessibility and seed regions. Bioinformatics **24:**2849-2856.
12. **Kery MB, Feldman M, Livny J, Tjaden B.** 2014. TargetRNA2: identifying targets of small regulatory RNAs in bacteria. Nucleic Acids Res **42:**W124-129.
